# Supplementary material for: Cytotoxic Activity, Topoisomerase I Inhibition and In Silico Studies of New Sesquiterpene-aryl Ester Derivatives of (-) Drimenol
Source: Molecules. 2023 May 8;28(9):3959. doi: 10.3390/molecules28093959 (PMC10179937; doi:10.3390/molecules28093959)
Supplement: Supplementary file 1 [file molecules-28-03959-s001.zip › molecules-2339130-supplementary.pdf]

molecules

MDPI

Supplementary Materials

Cytotoxic Activity, Topoisomerase I Inhibition and In Silico Studies of New Sesquiterpene-aryl Ester Derivatives of (-) Drimenol

Ileana Araque [1], Javiera Ramírez [1], Rut Vergara [2], Jaime Mella [3,4], Pablo Aránguiz [5], Luis Espinoza [6], Waleska Vera [1,4], Iván Montenegro [4,7], Cristian O. Salas [8], Joan Villena [2] and Mauricio A. Cuellar [1,4,*]

[1] Facultad de Farmacia, Escuela de Química y Farmacia, Universidad de Valparaíso, Av. Gran Bretaña 1093, Valparaíso 2340000, Chile
[2] Centro de Investigaciones Biomédicas, Escuela de Medicina, Facultad de Medicina, Universidad de Valparaíso, Viña del Mar 2520000, Chile
[3] Instituto de Química y Bioquímica, Facultad de Ciencias, Universidad de Valparaíso, Valparaíso 2340000, Chile
[4] Centro de Investigación Farmacopea Chilena (CIFAR), Universidad de Valparaíso, Valparaíso 2340000, Chile
[5] Escuela de Química y Farmacia, Facultad de Medicina, Universidad Andrés Bello, Viña del Mar 2520000, Chile
[6] Departamento de Química, Universidad Técnica Federico Santa María, Avenida España 1680, Valparaíso 2340000, Chile
[7] Facultad de Medicina, Escuela de Obstetricia y Puericultura, Universidad de Valparaíso, Angamos 655, Reñaca, Viña del Mar 2520000, Chile
[8] Departamento de Química Orgánica, Facultad de Química y de Farmacia, Pontificia Universidad Católica de Chile, Avenida Vicuña Mackenna 4860, Macul, Santiago de Chile 7820436, Chile
* Correspondence: mauricio.cuellar@uv.cl; Tel.: +56-32-2508439

Index

^{1}H,^{13}C NMR and ^{13}C-DEPT 135 of selected compounds ……………………………………………. page 02–17
HRMS of selected compounds………………………………………………………….……….………. page 18–23
Molecular Docking and Molecular Dynamics results………………………………………………. page 24–26

^{1}H NMR for drimenal

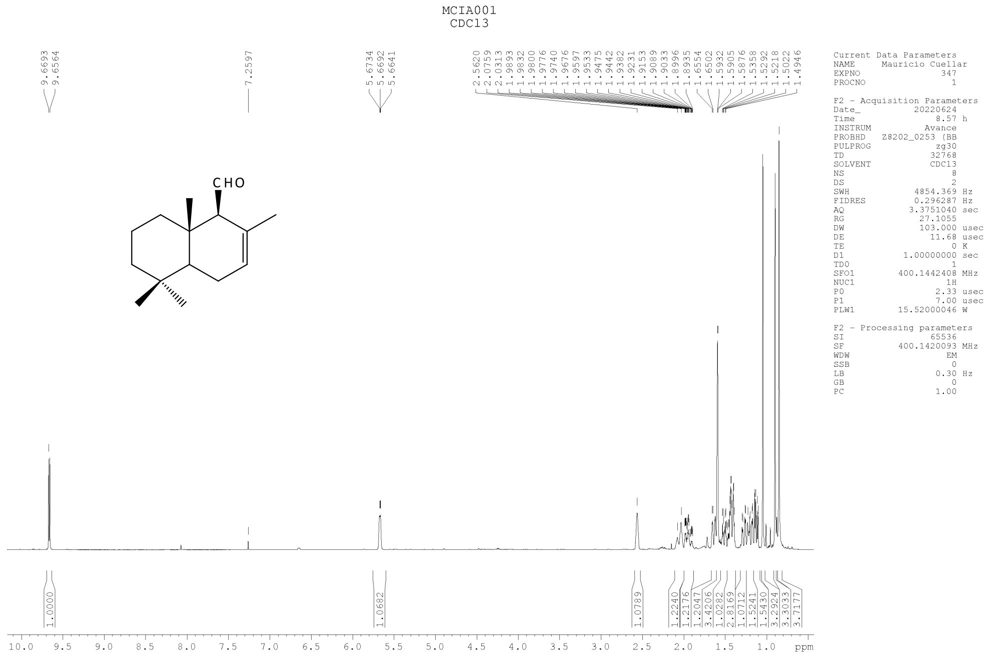

^{13}C NMR for drimenal

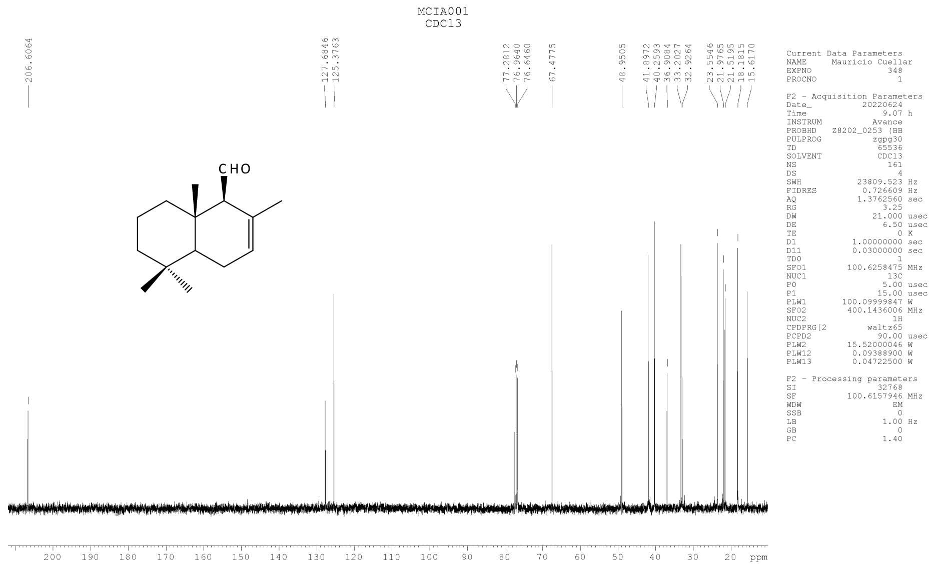

^{13}C-DEPT 135 NMR for drimenal

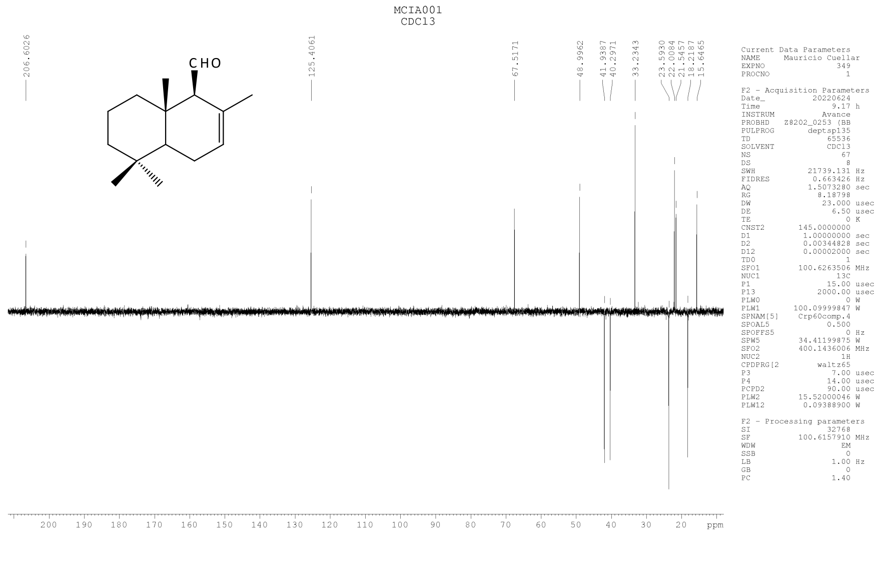

^{1}H NMR for drimenic acid

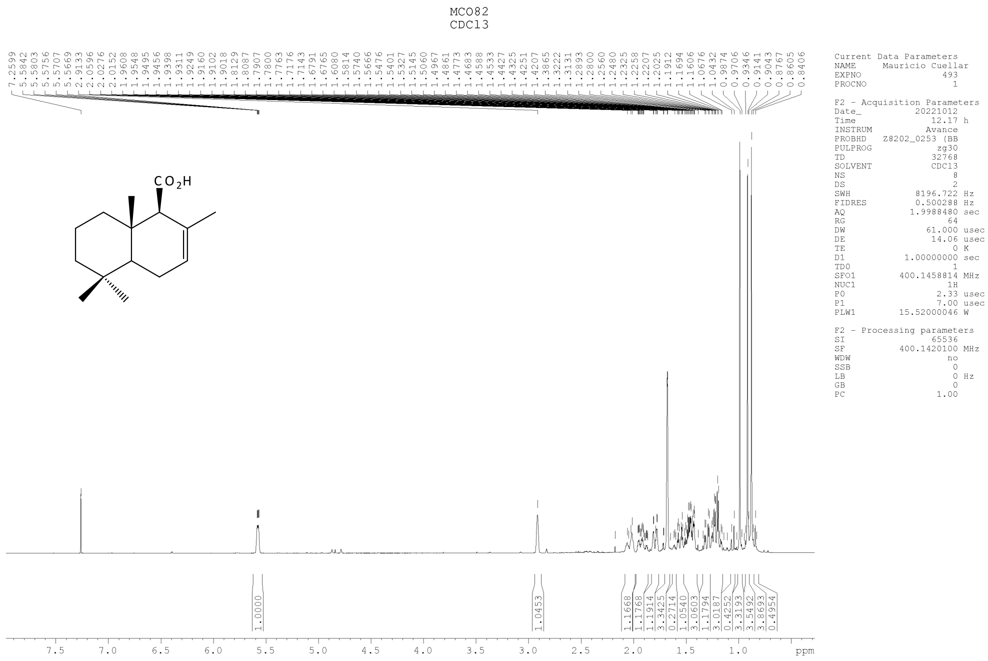

^{13}C NMR for drimenic acid

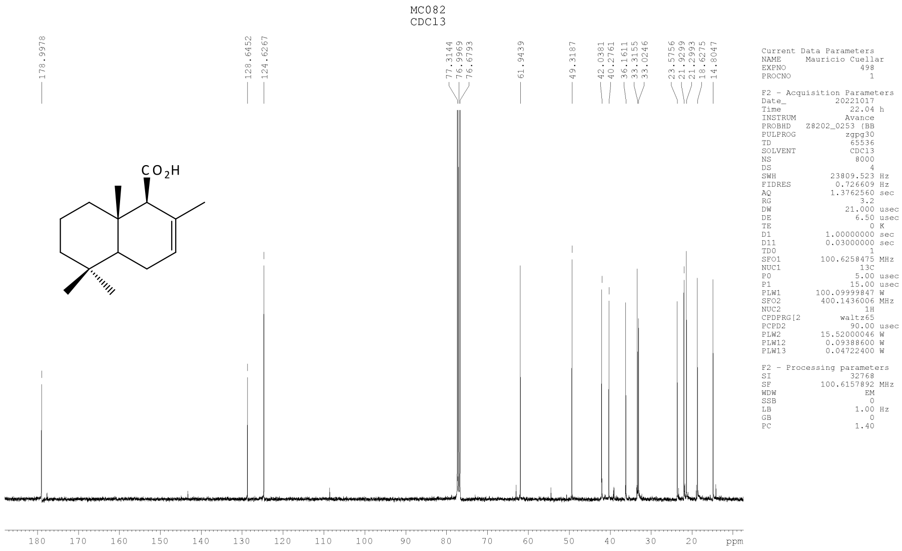

^{13}C-DEPT 135 NMR for drimenic acid

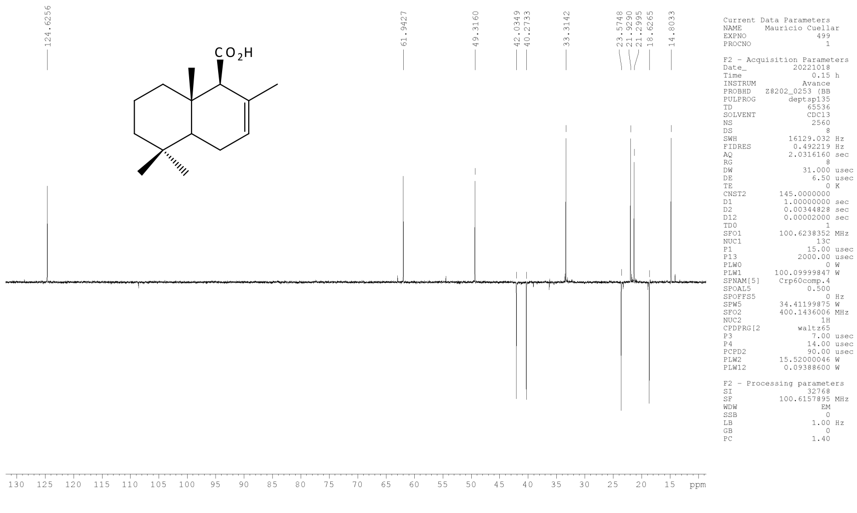

^{1}H NMR for 6a

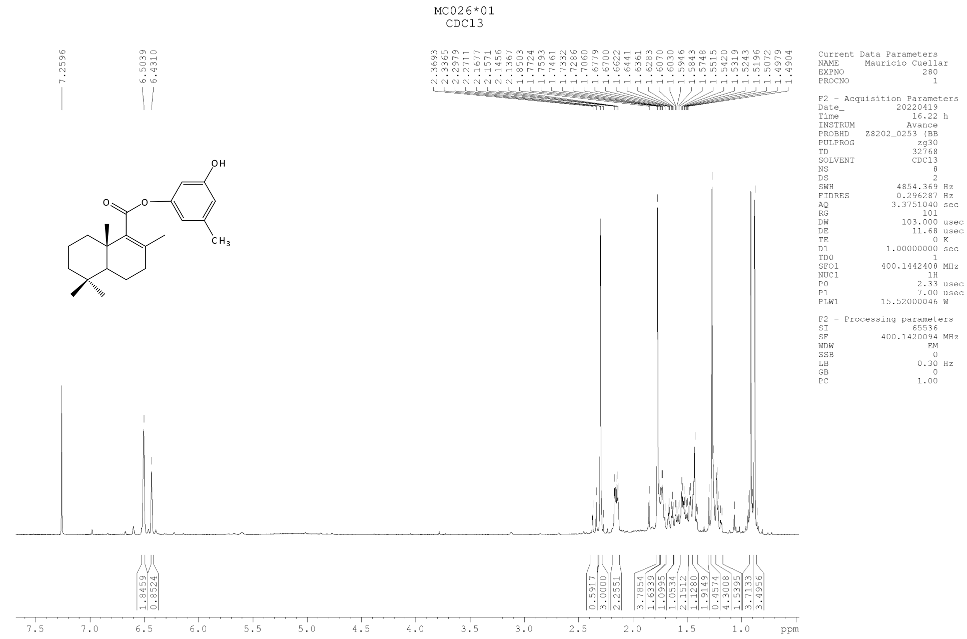

^{13}C NMR for 6a

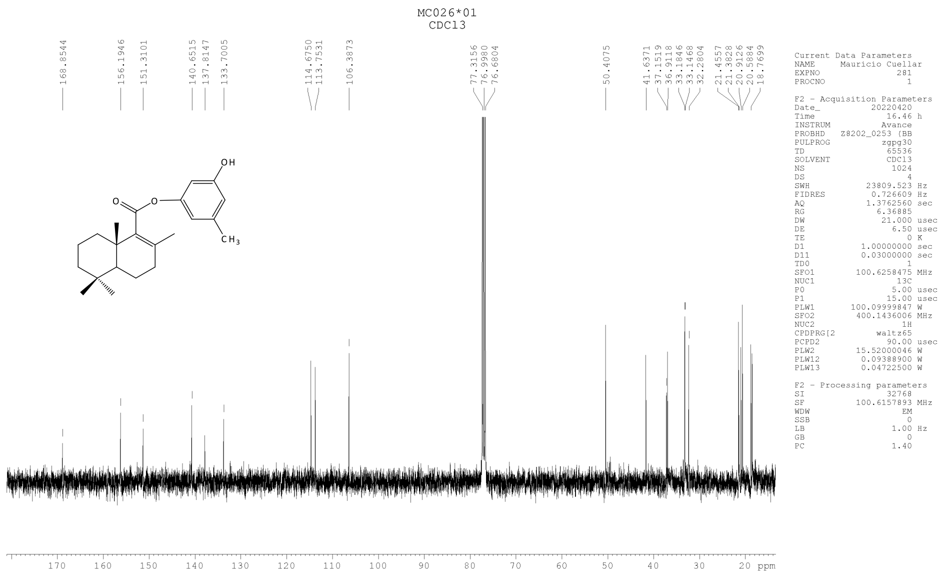

^{13}C-DEPT 135 NMR for 6a

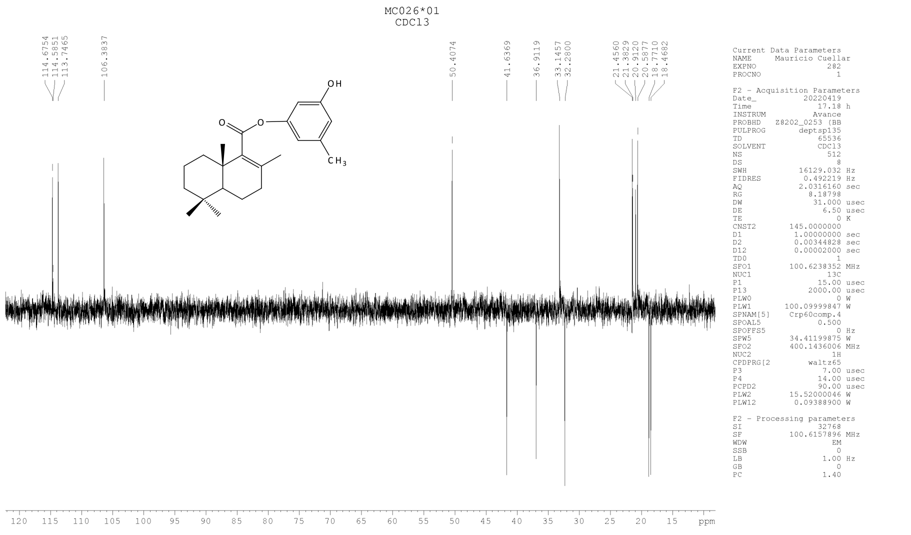

^{1}H NMR for 6b

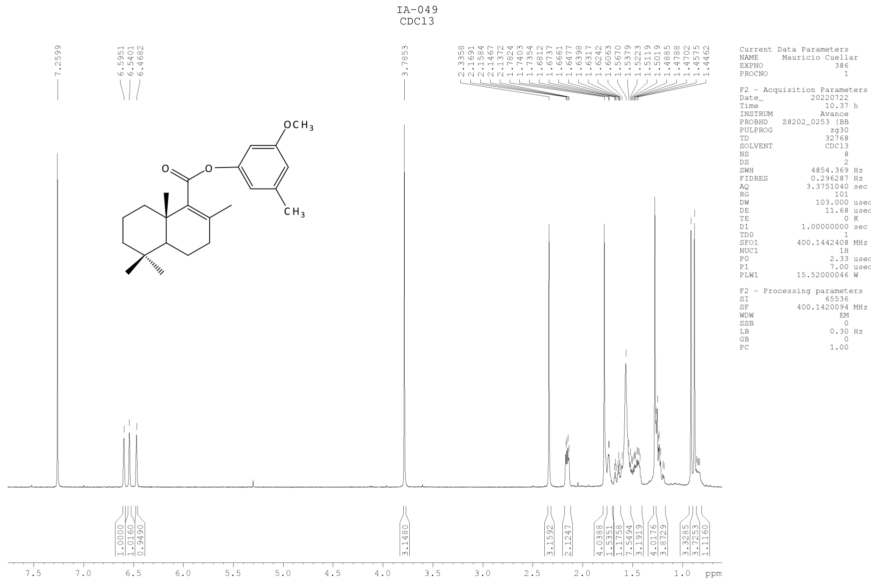

^{13}C NMR for 6b

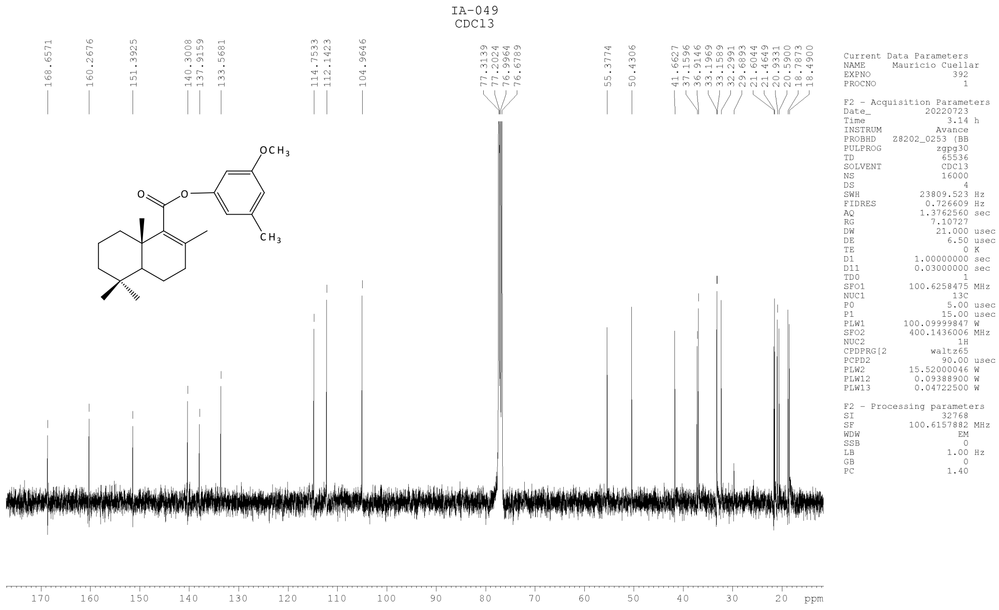

^{13}C-DEPT 135 NMR for 6b

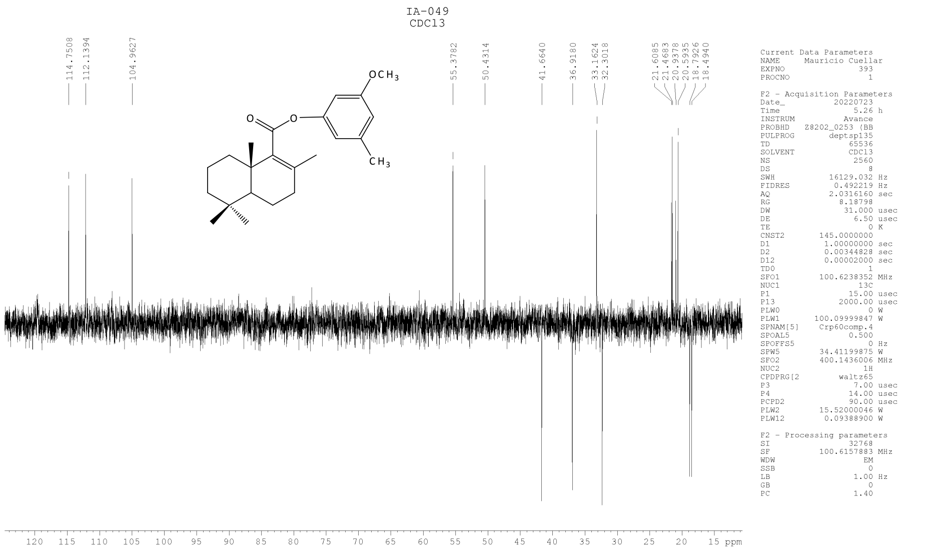

^{1}H NMR for 6c

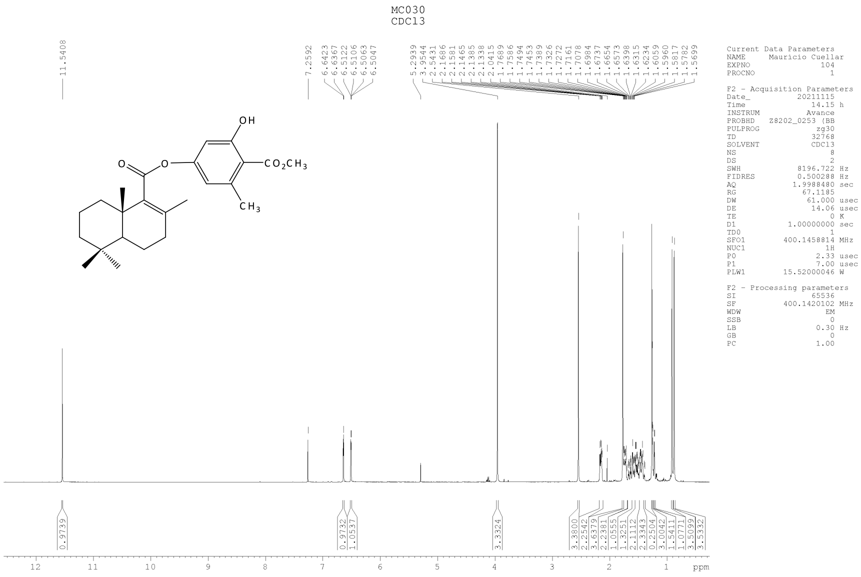

^{13}C NMR for 6c

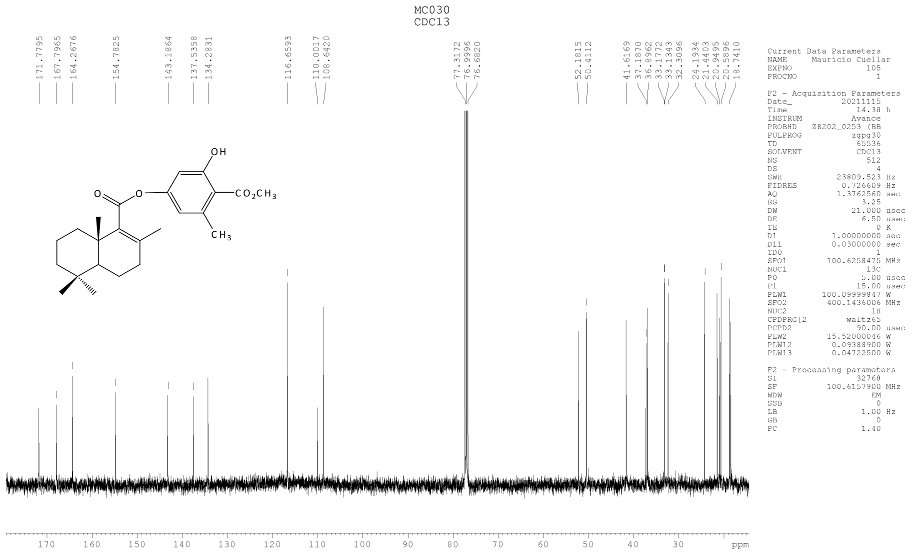

^{13}C-DEPT 135 NMR for 6c

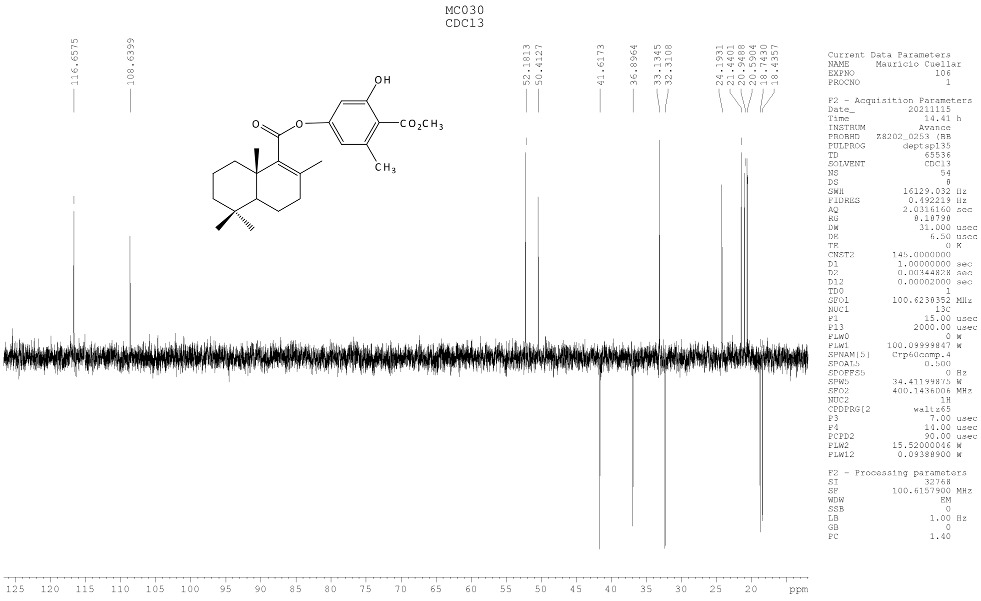

^{1}H NMR for 6d

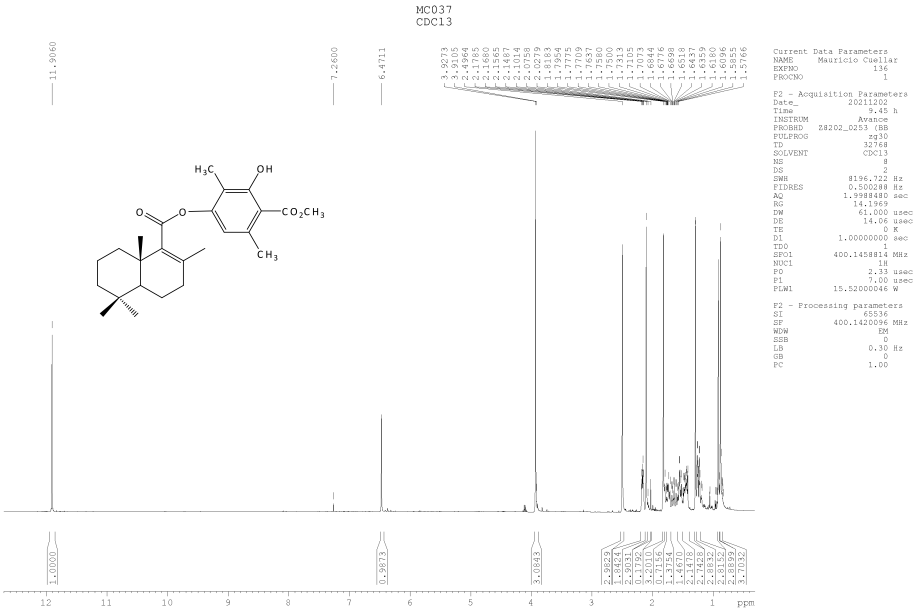

^{13}C NMR for 6d

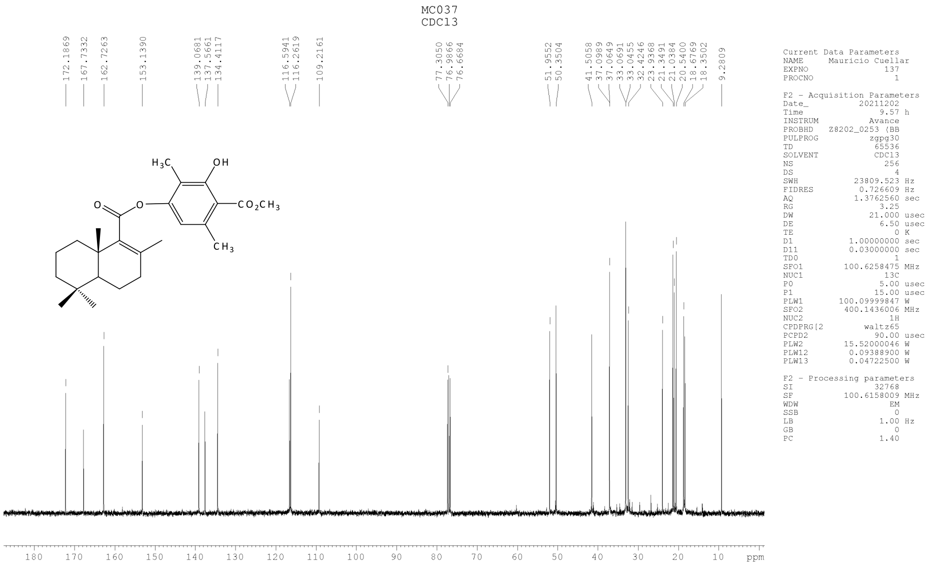

^{13}C-DEPT 135 NMR for 6d

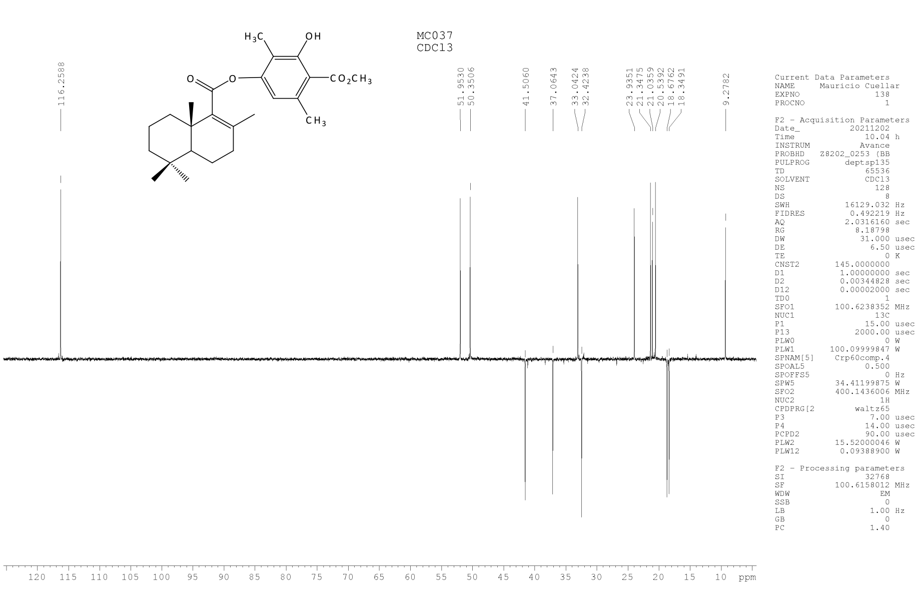

^{1}H NMR for 6e

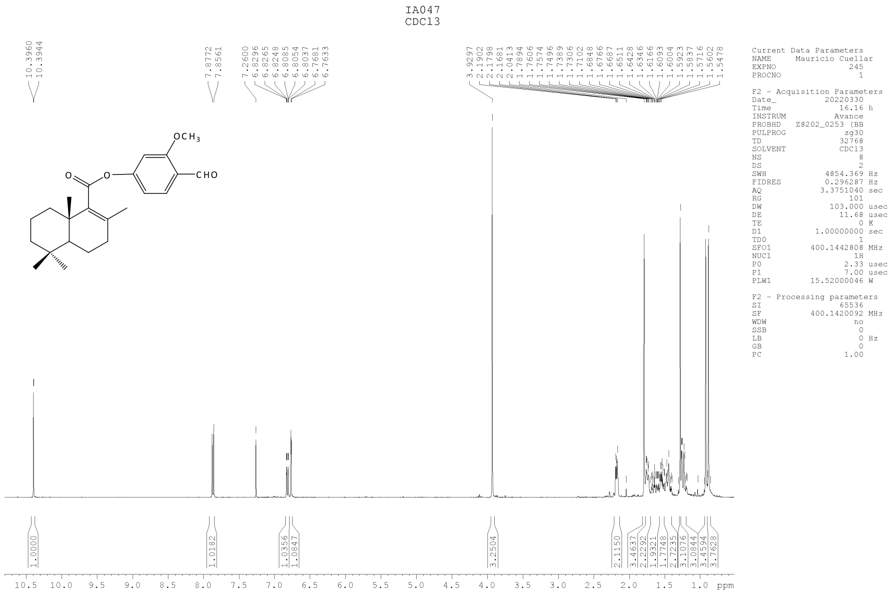

^{13}C NMR for 6e

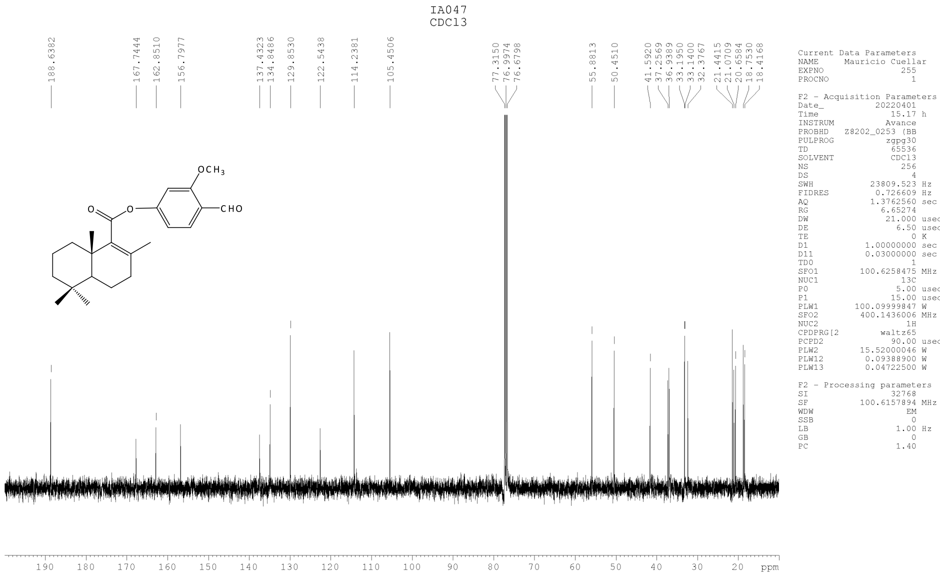

^{13}C-DEPT 135 NMR for 6e

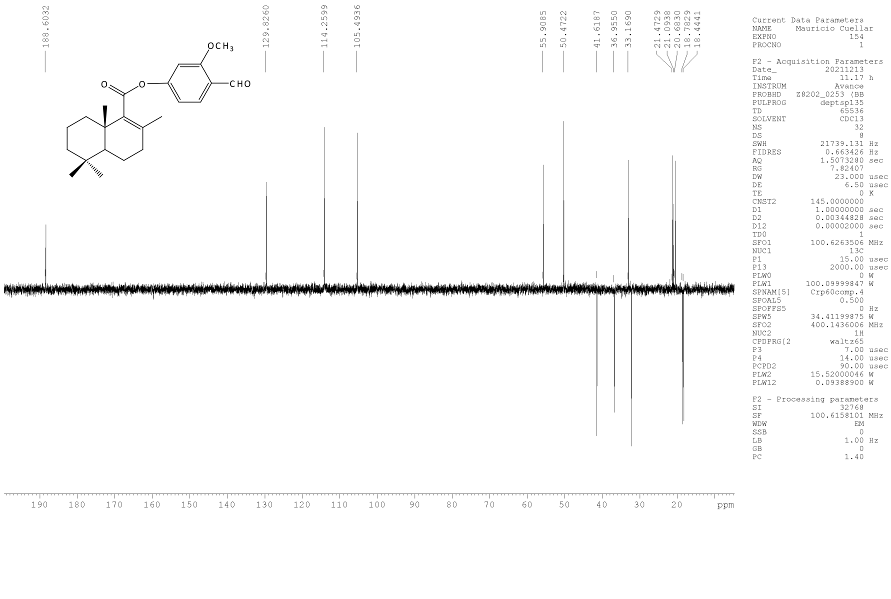

^{1}H NMR for 6f

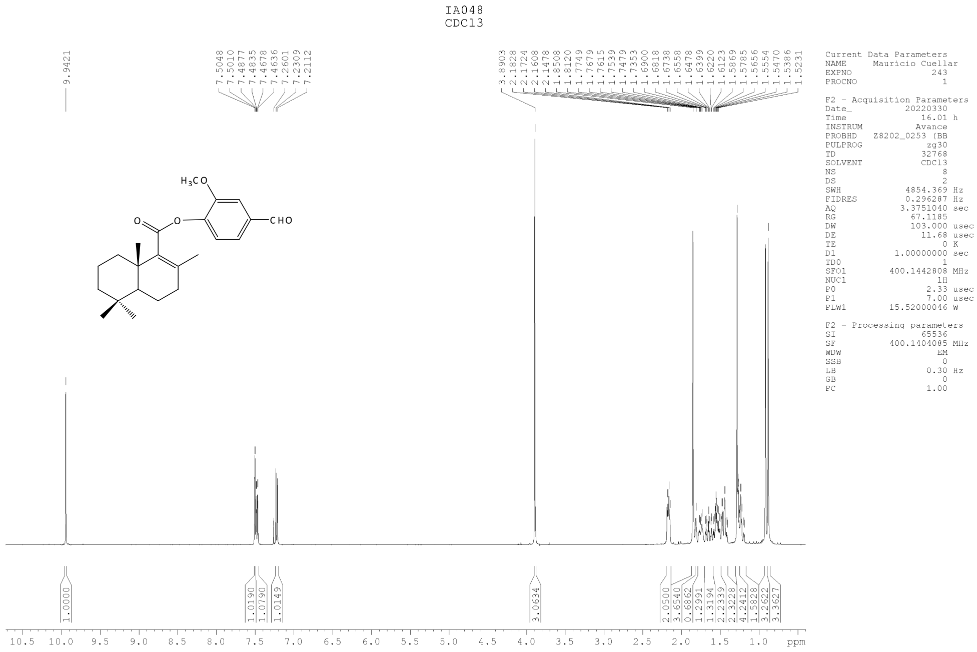

^{13}C NMR for 6f

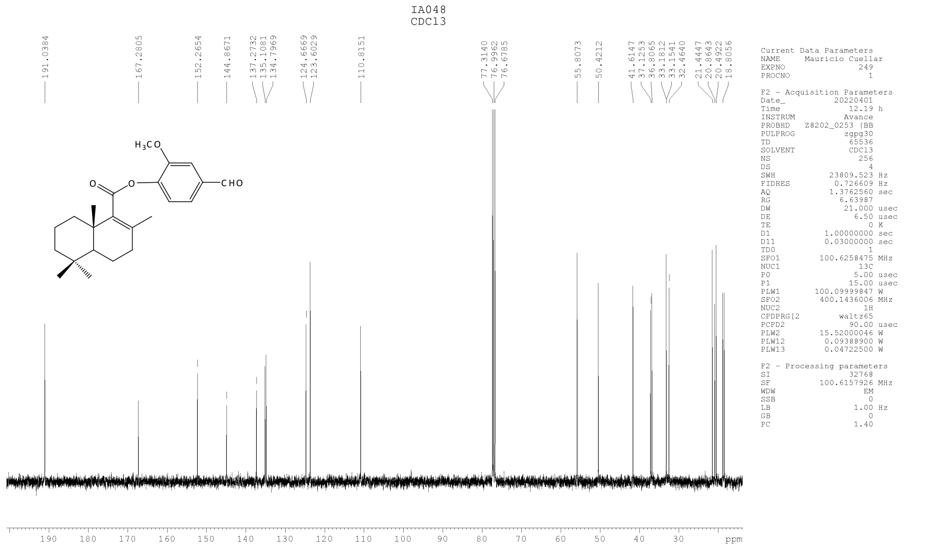

^{13}C-DEPT 135 NMR for 6f

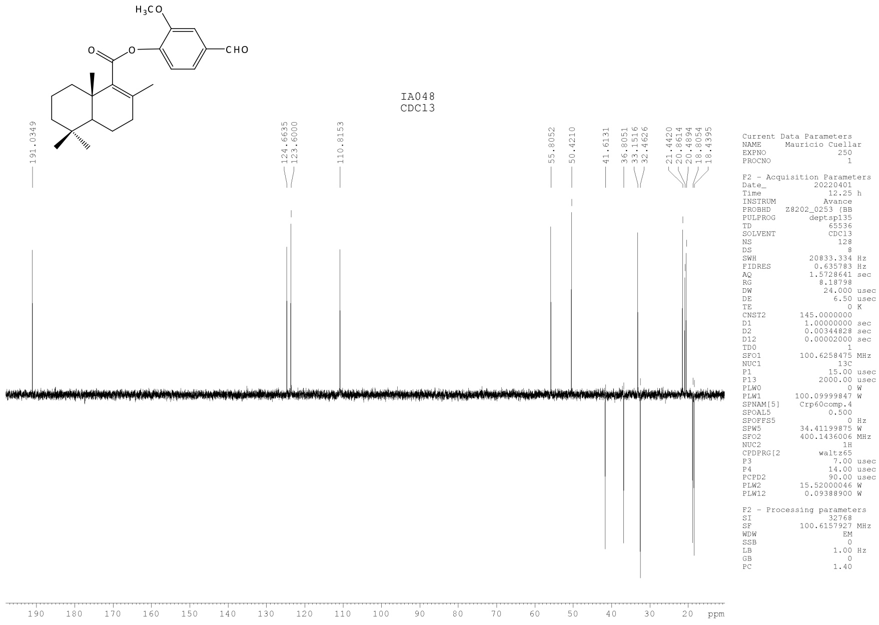

^{1}H NMR for 8a

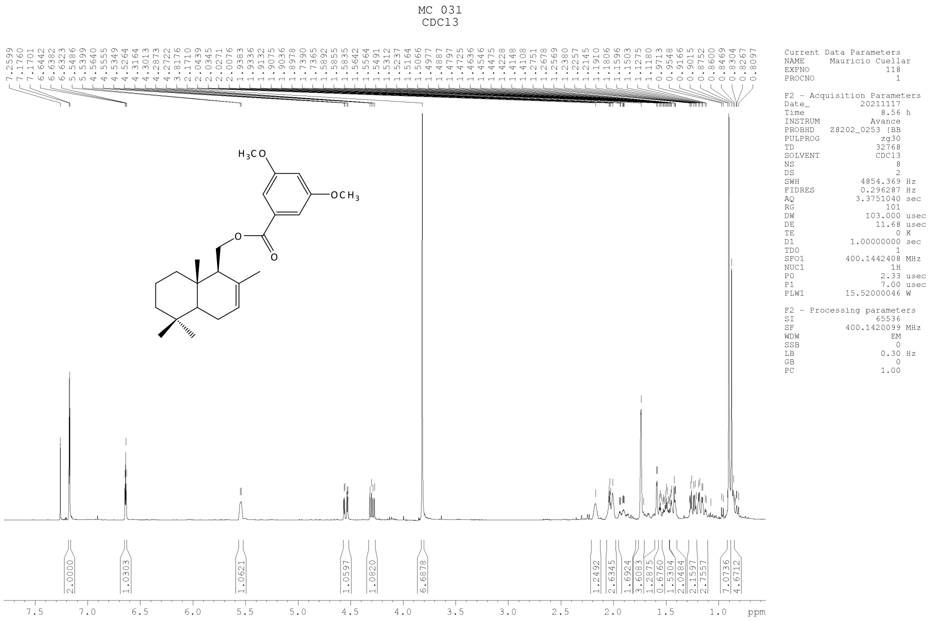

^{13}C NMR for 8a

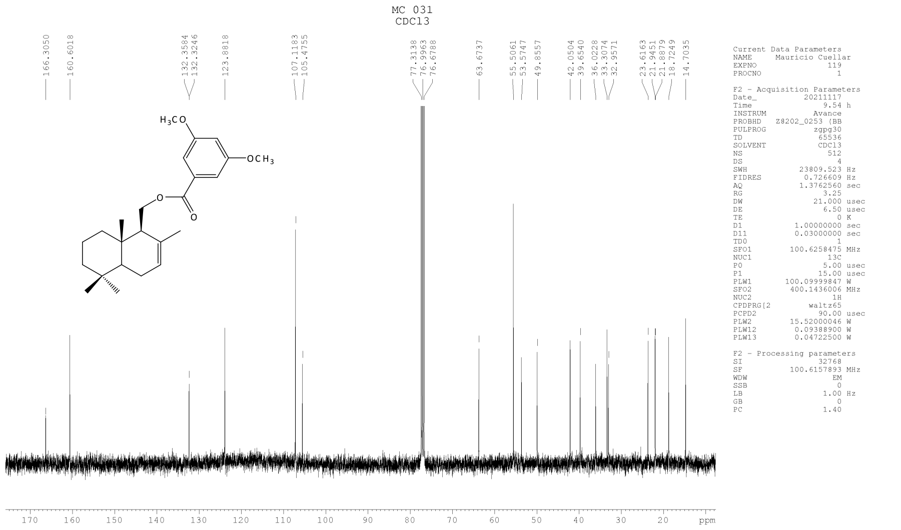

^{13}C-DEPT 135 NMR for 8a

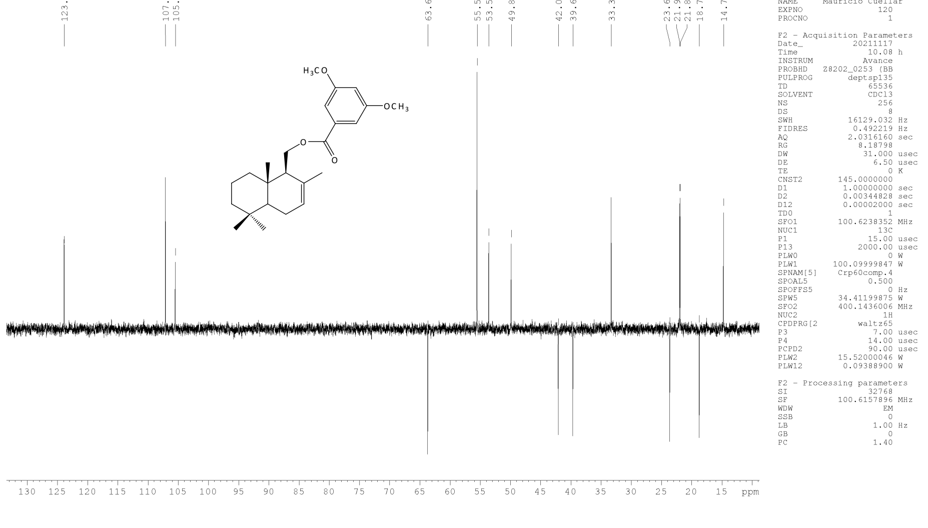

^{1}H NMR for 8b

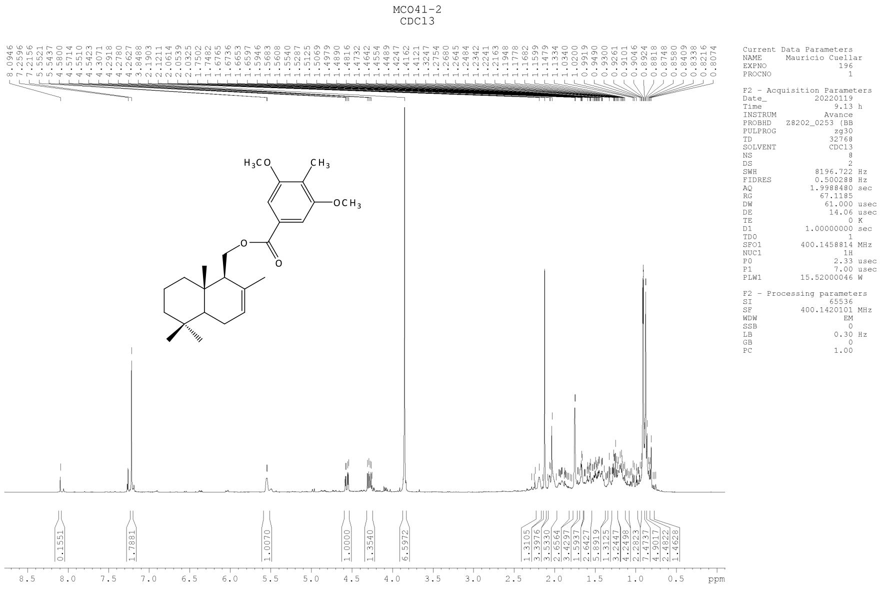

^{13}C NMR for 8b

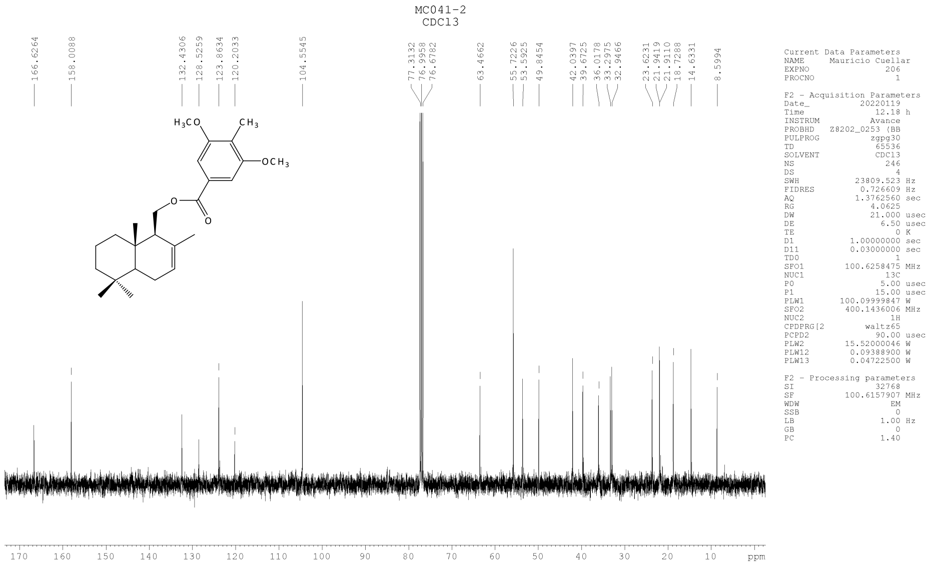

^{13}C-DEPT 135 NMR for 8b

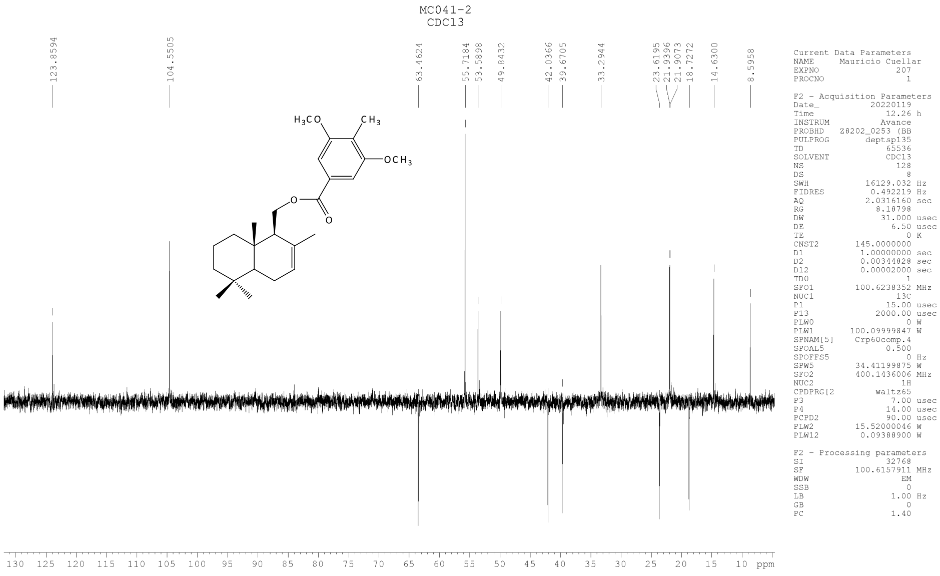

^{1}H NMR for 8c

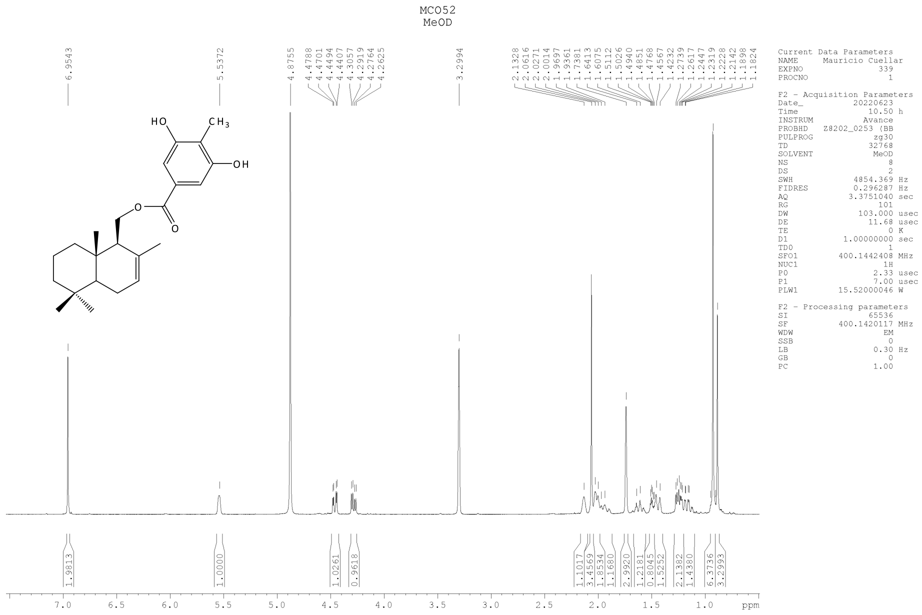

^{13}C NMR for 8c

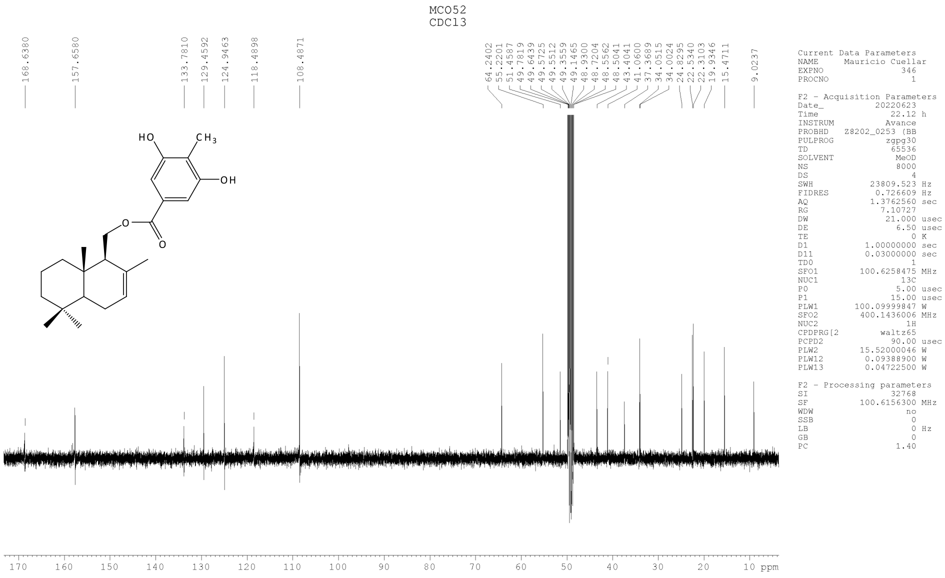

^{13}C-DEPT 135 NMR for 8c

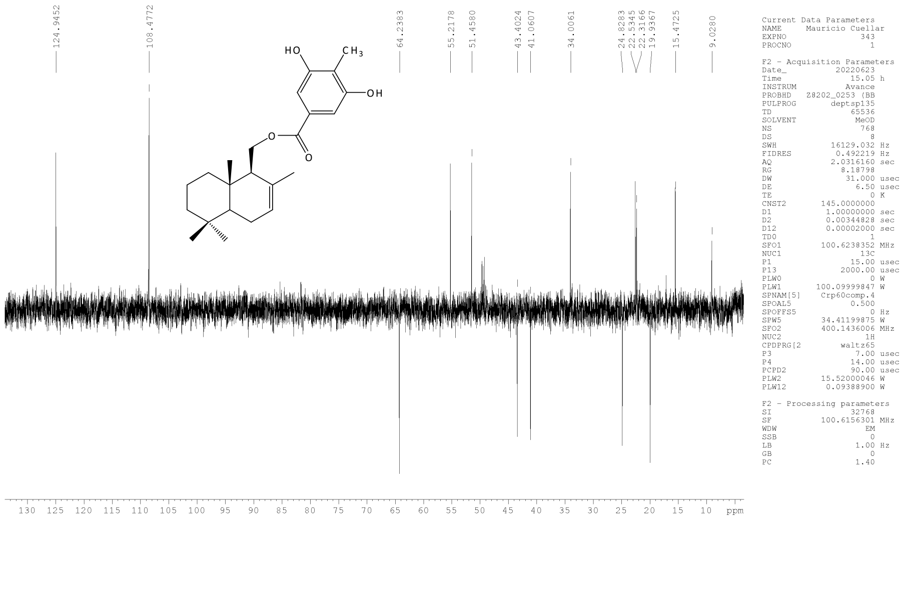

^{1}H NMR for 8d

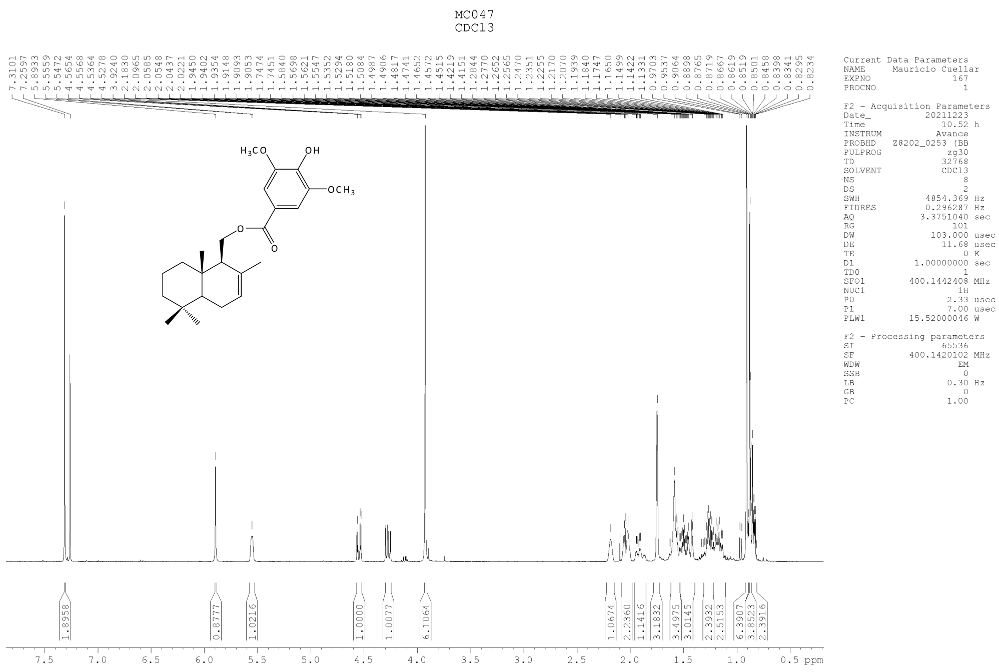

^{13}C NMR for 8d

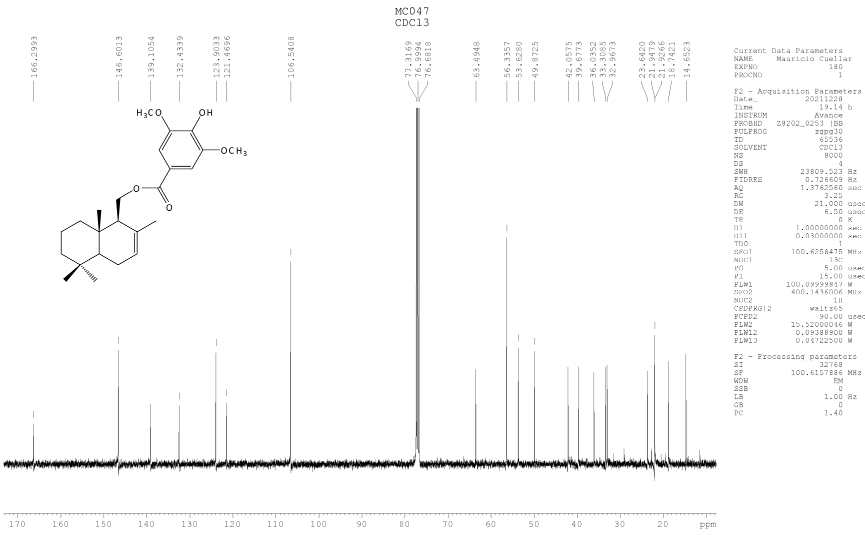

^{13}C-DEPT 135 NMR for 8d

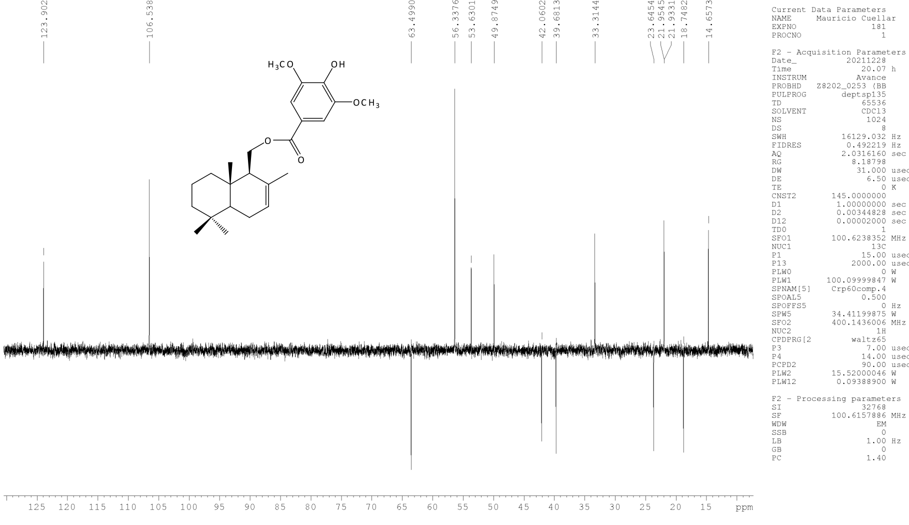

^{1}H NMR for 8e

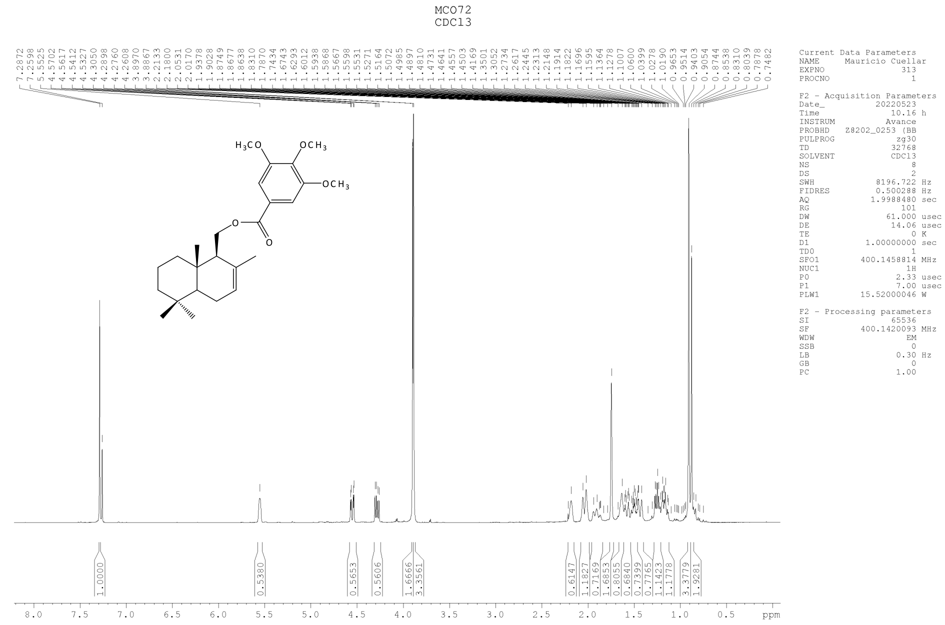

^{13}C NMR for 8e

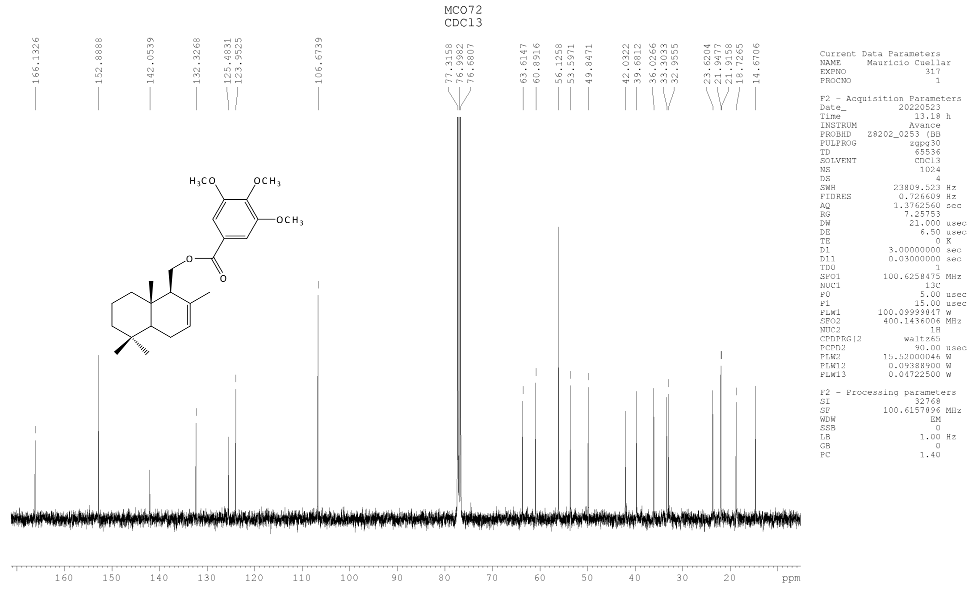

^{13}C-DEPT 135 NMR for 8e

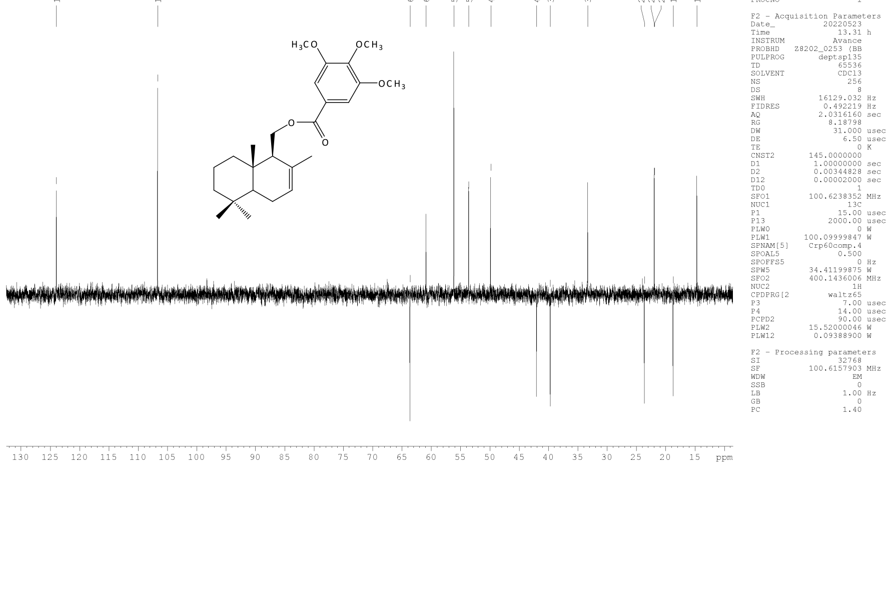

^{1}H NMR for 8f

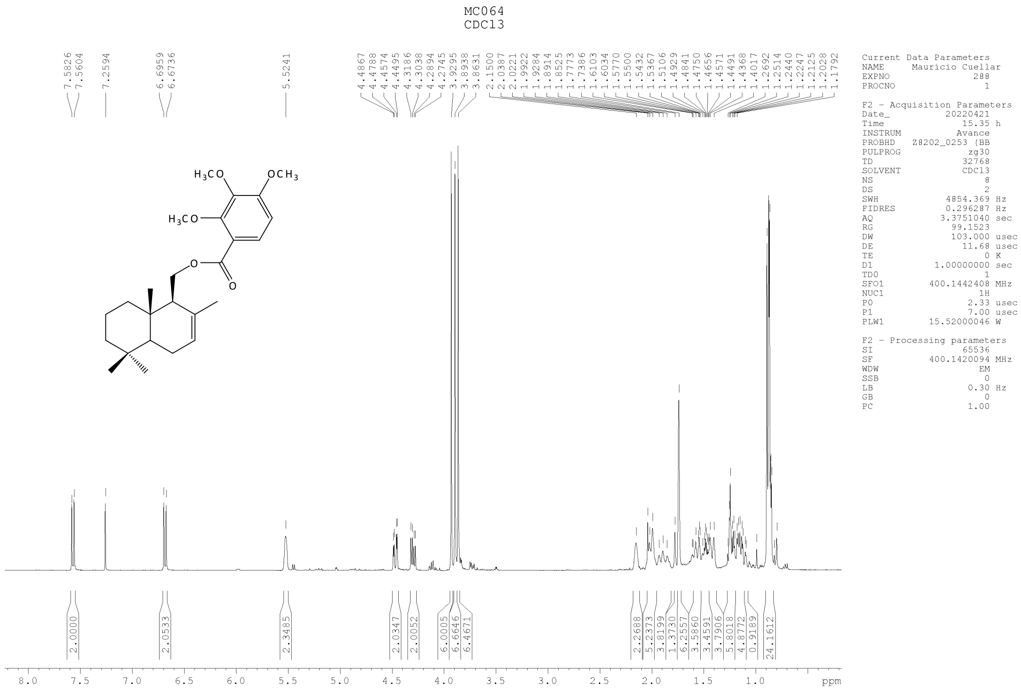

^{13}C NMR for 8f

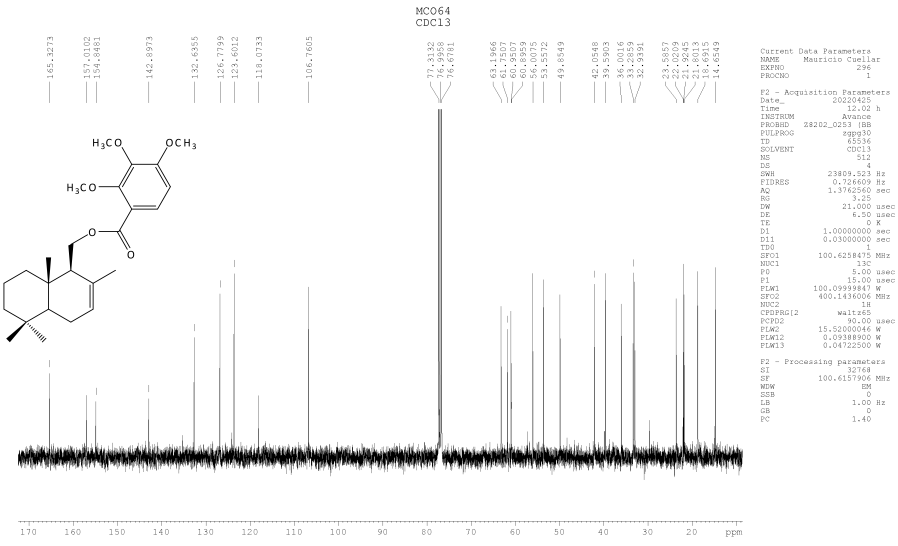

^{13}C-DEPT 135 NMR for 8f

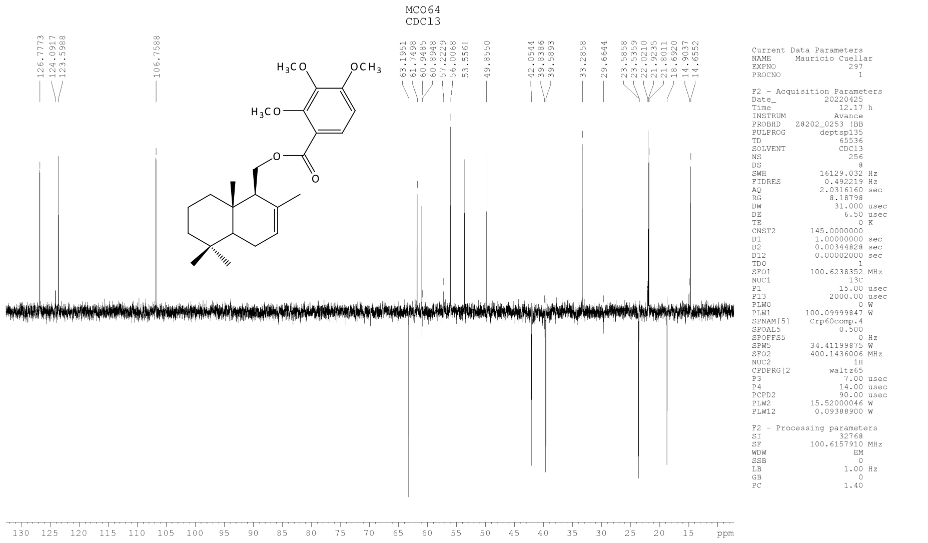

^{1}H NMR for 8g

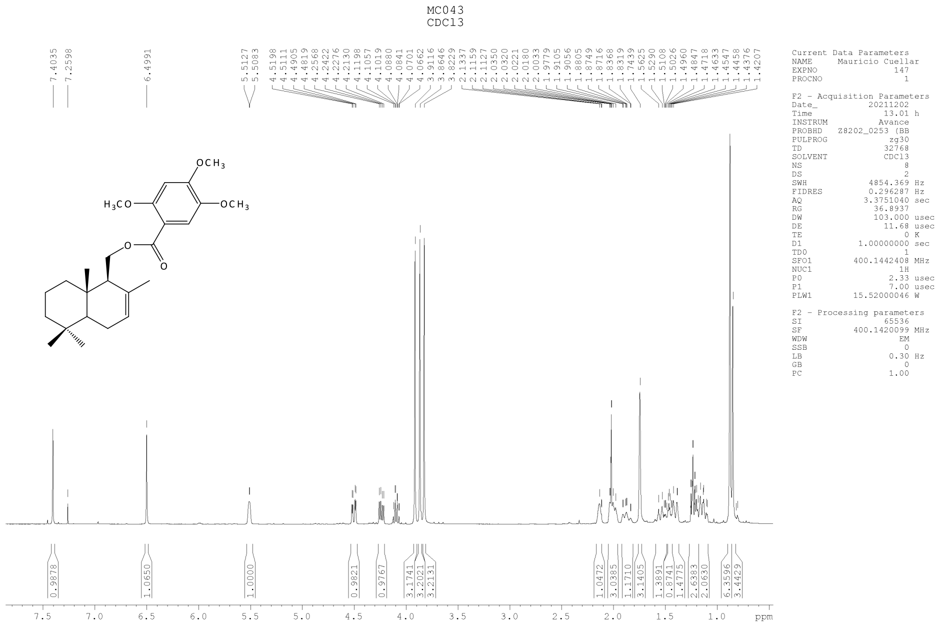

^{13}C NMR for 8g

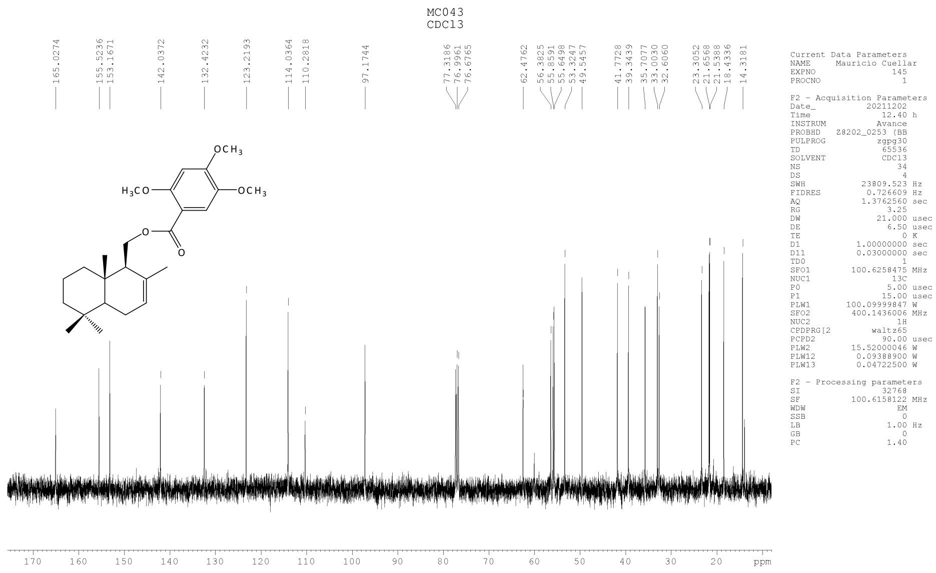

^{13}C-DEPT 135 NMR for 8g

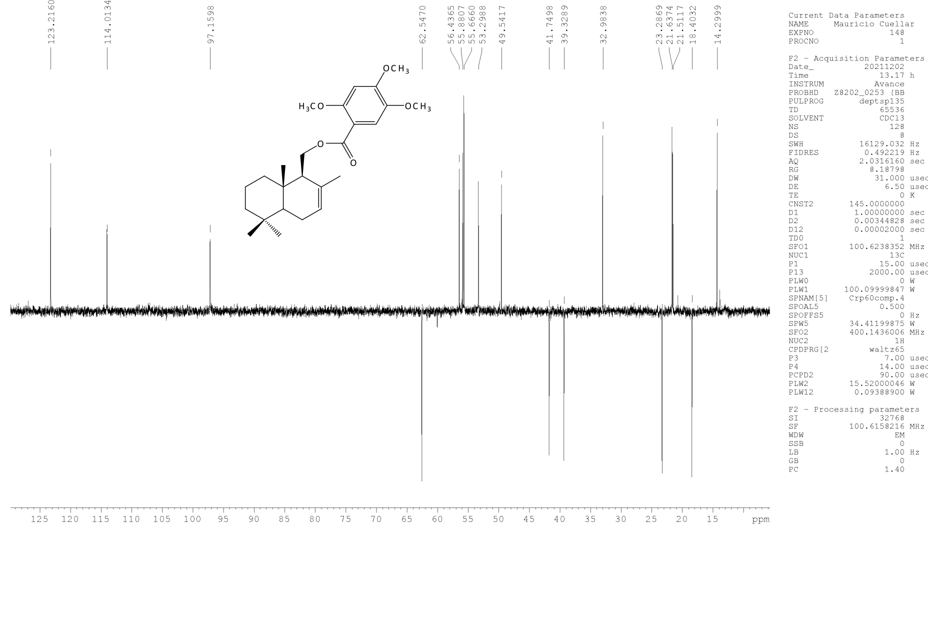

HRMS for **6a**

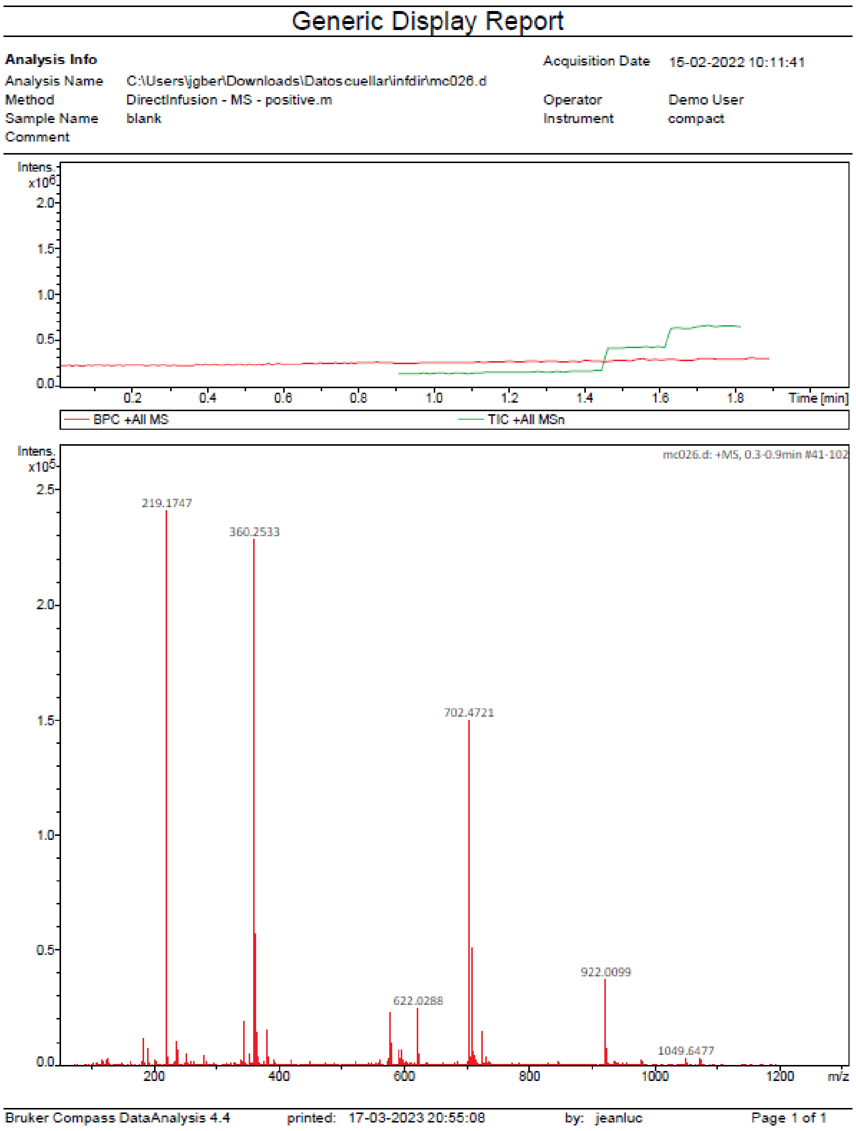

HRMS for **6b**

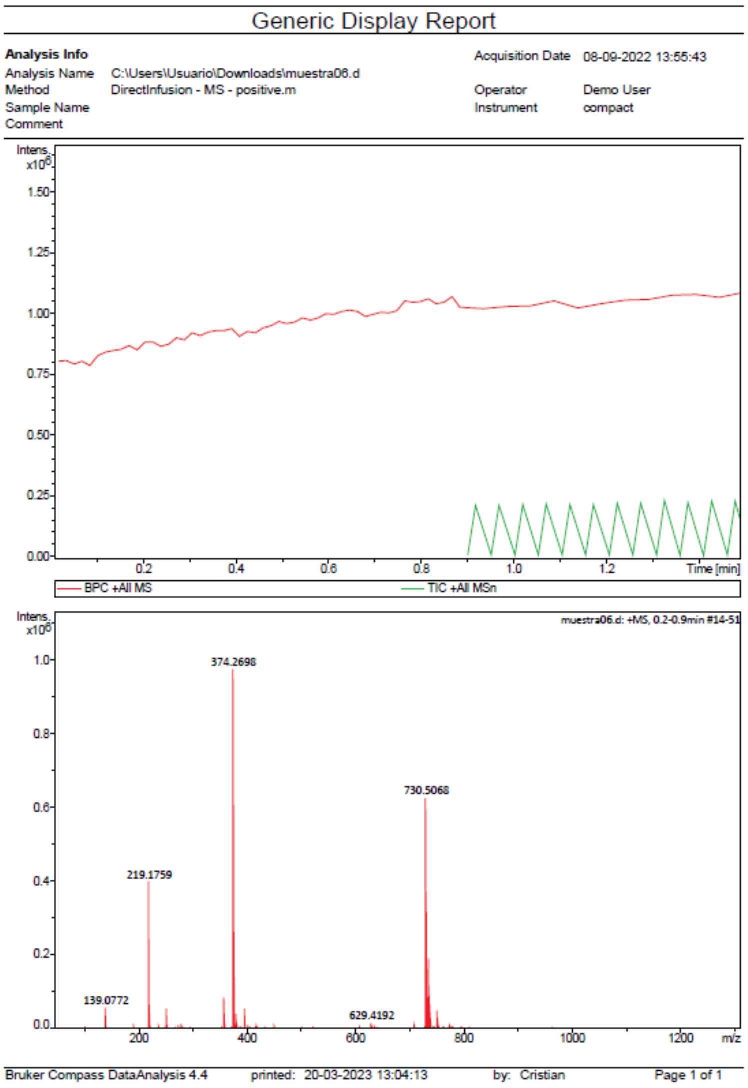

HRMS for **6c**

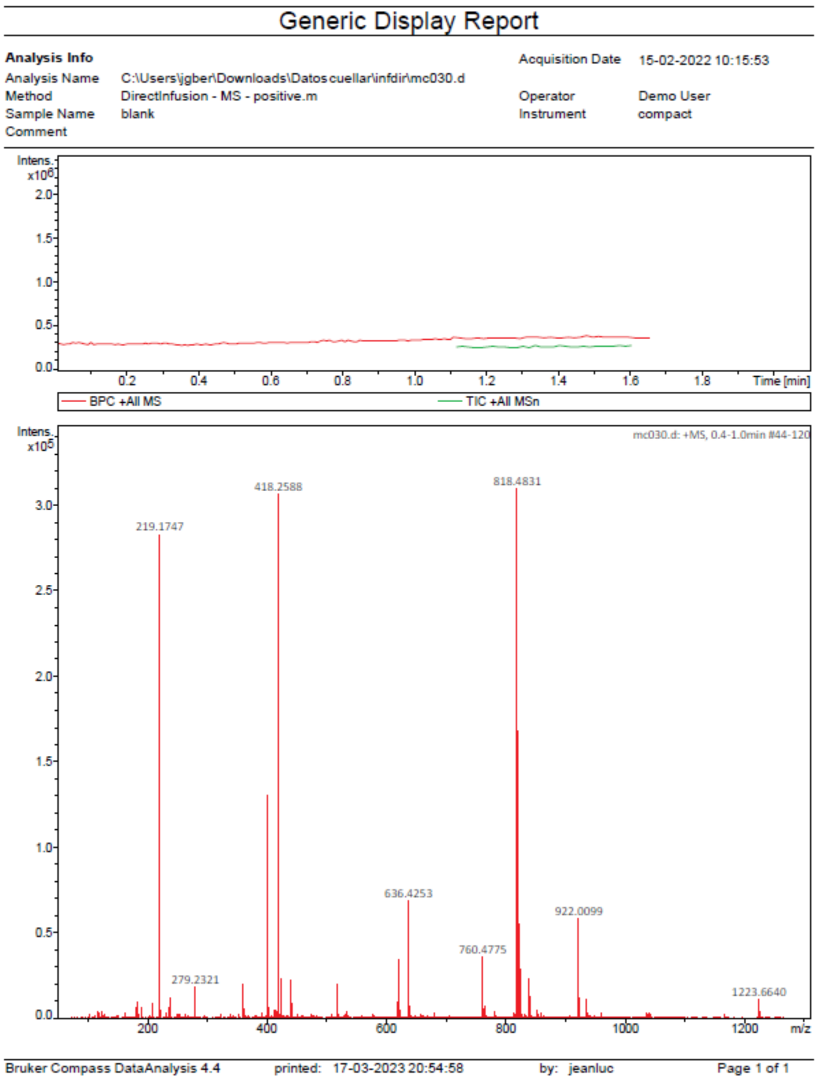

HRMS for **6d**

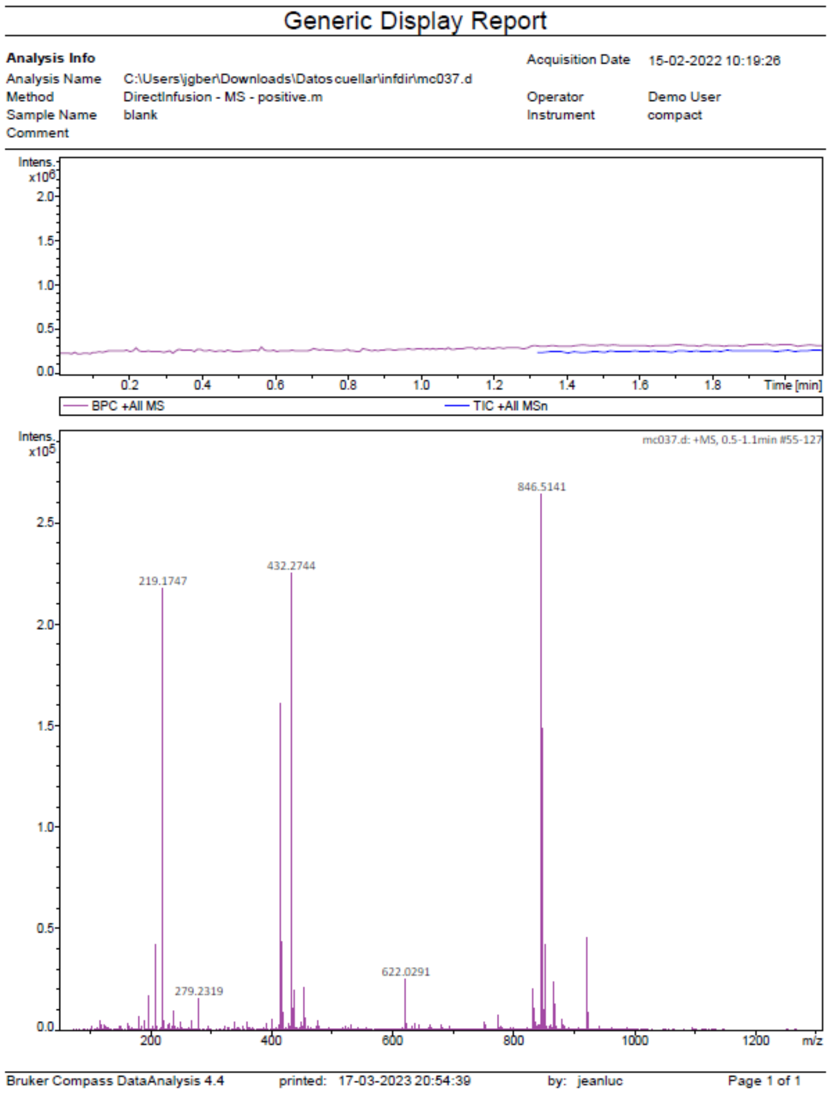

HRMS for **6e**

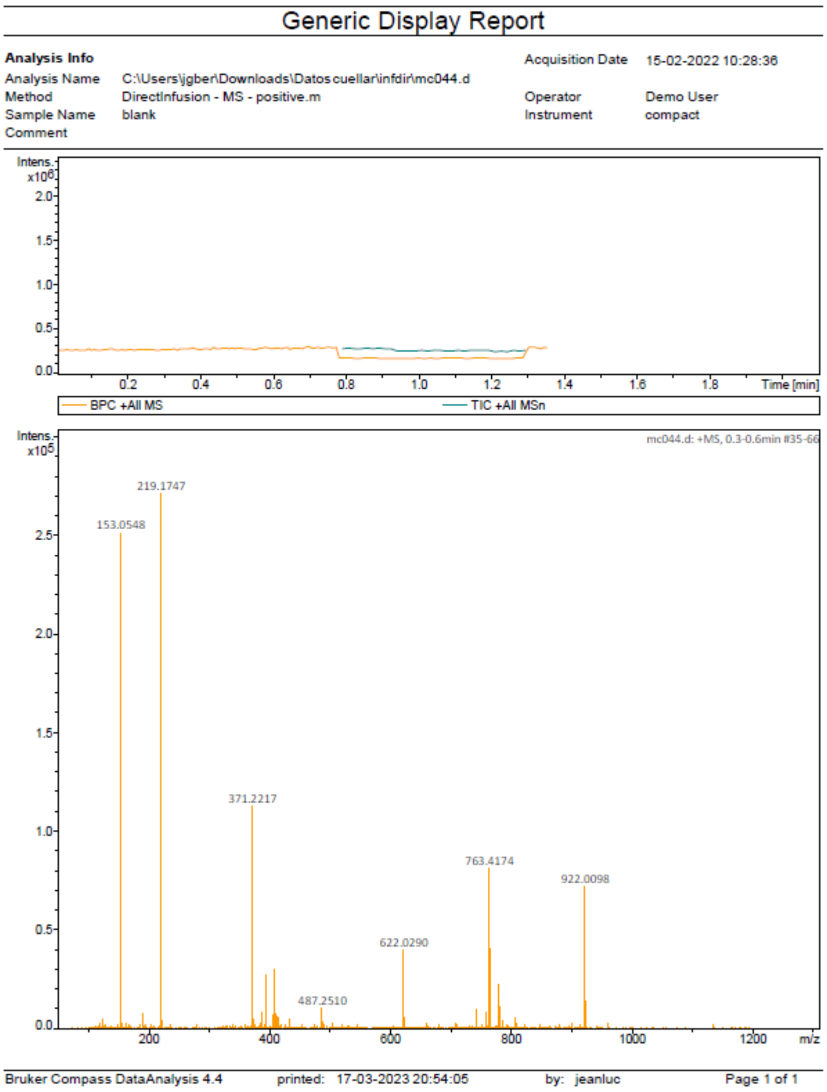

HRMS for **6f**

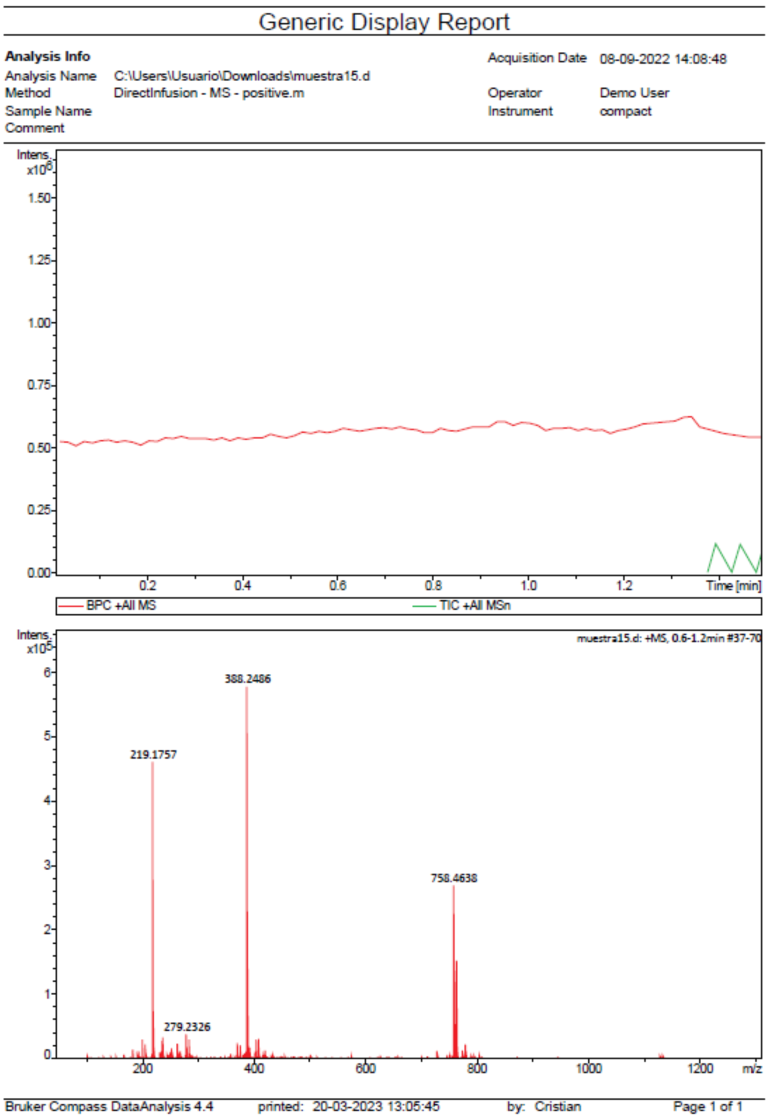

HRMS for **8a**

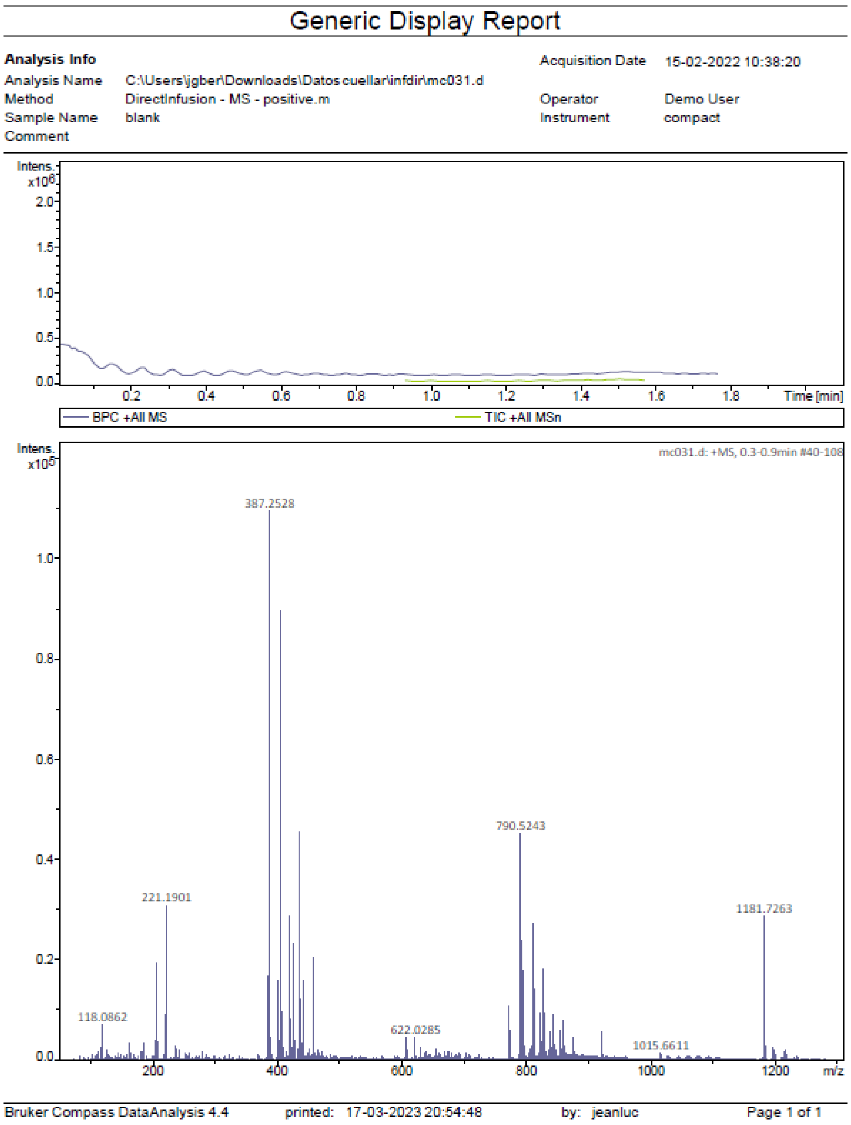

HRMS for **8b**

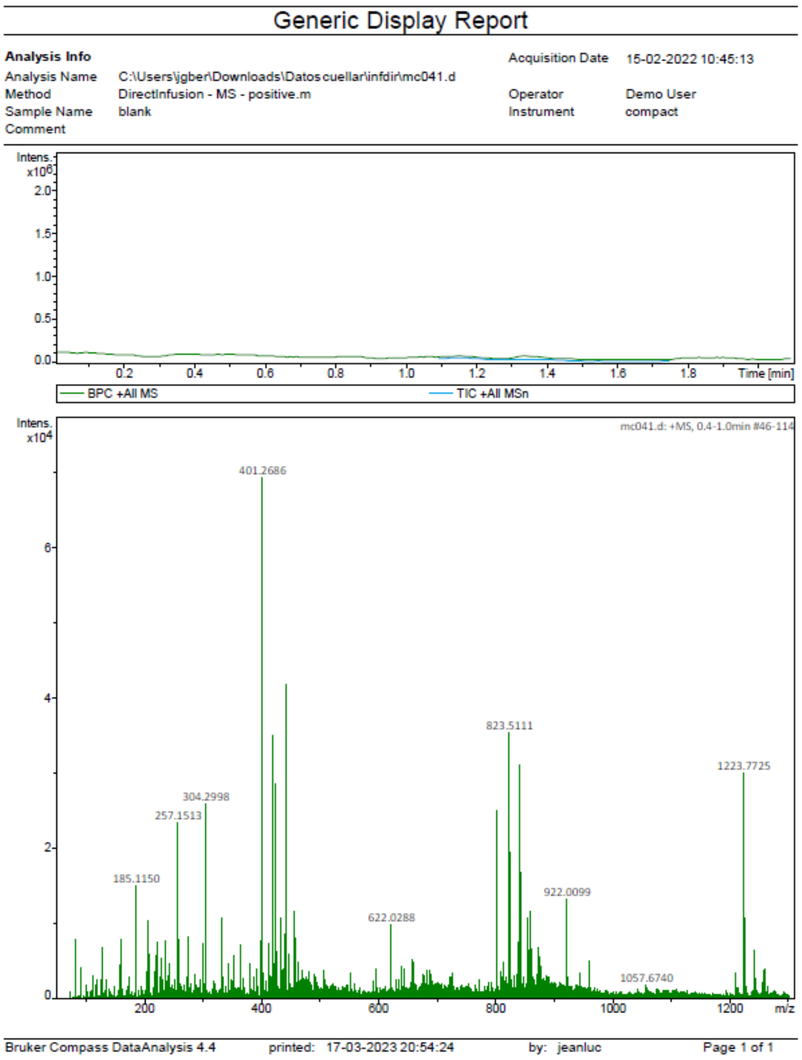

HRMS for **8c**

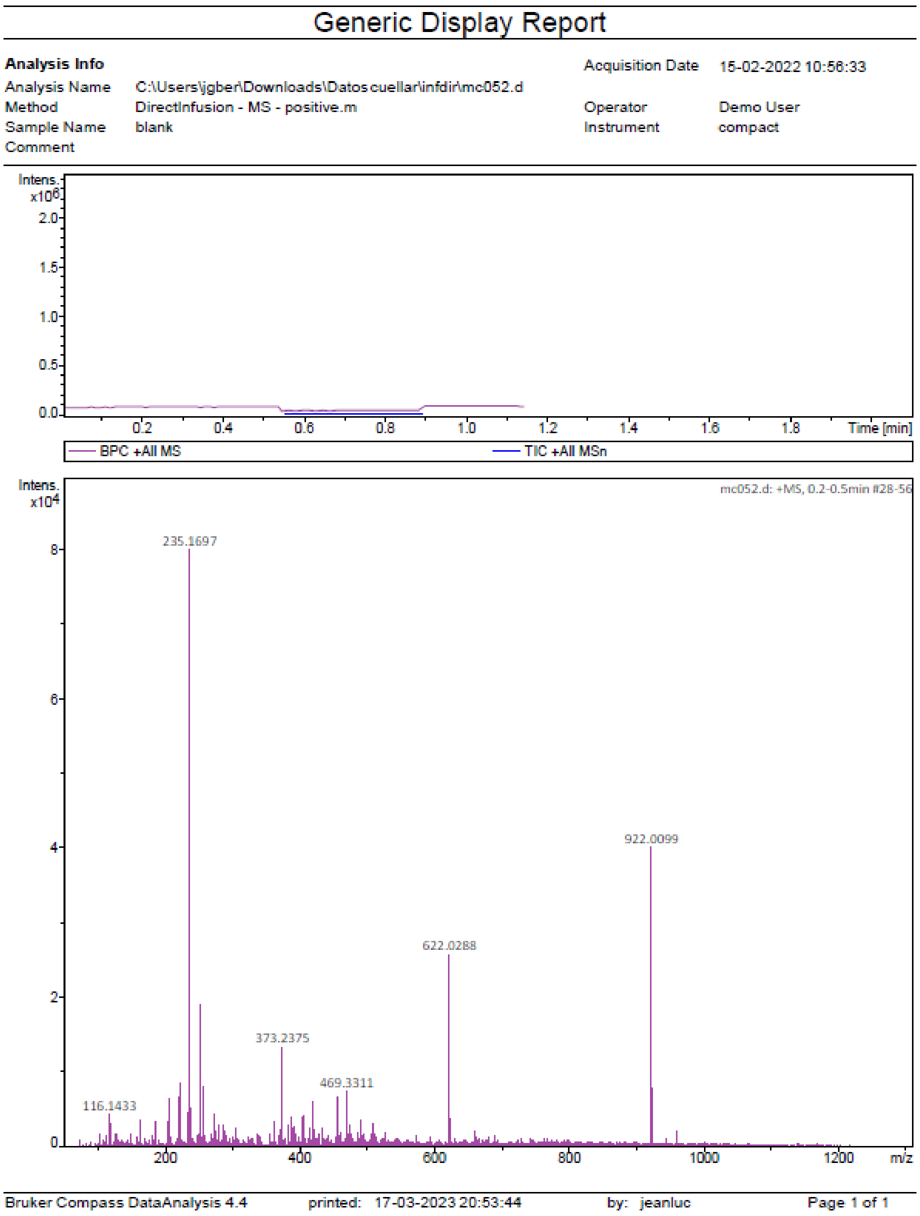

HRMS for **8d**

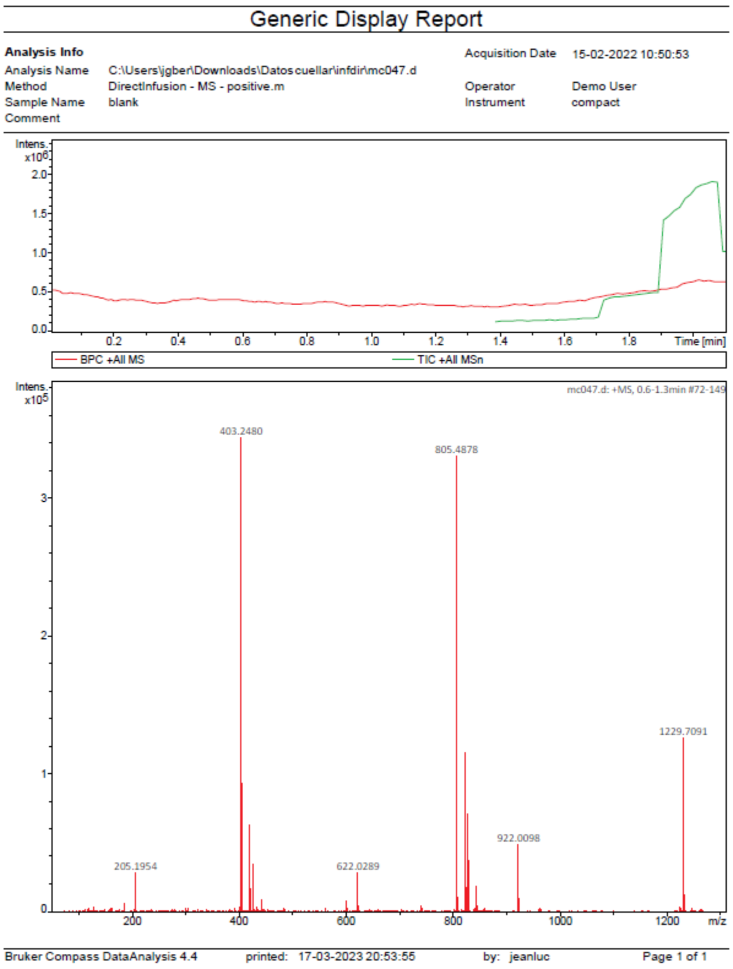

HRMS for **8e**

Generic Display Report

Analysis Info			
Analysis Name	C:\Users\Usuario\Downloads\muestra13.d	Acquisition Date	08-09-2022 14:03:03
Method	DirectInfusion - MS - positive.m	Operator	Demo User
Sample Name		Instrument	compact
Comment			

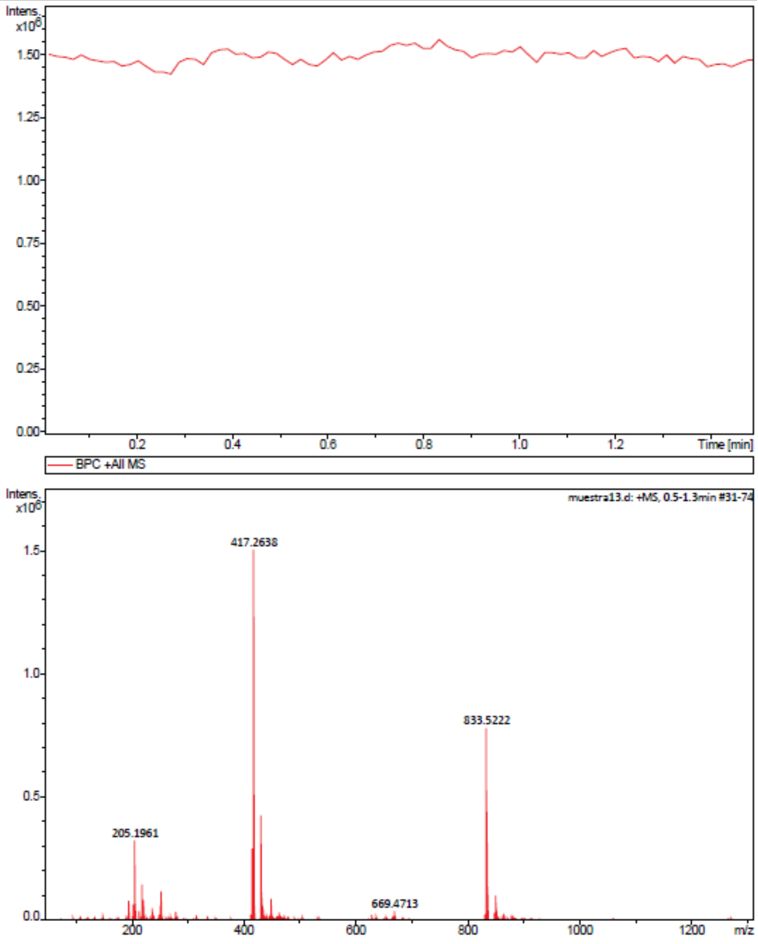

Bruker Compass DataAnalysis 4.4 printed: 20-03-2023 13:06:15 by: Cristian Page 1 of 1

HRMS for **8f**

Generic Display Report

Analysis Info			
Analysis Name	C:\Users\Usuario\Downloads\muestra12.d	Acquisition Date	08-09-2022 12:39:13
Method	DirectInfusion - MS - positive.m	Operator	Demo User
Sample Name		Instrument	compact
Comment			

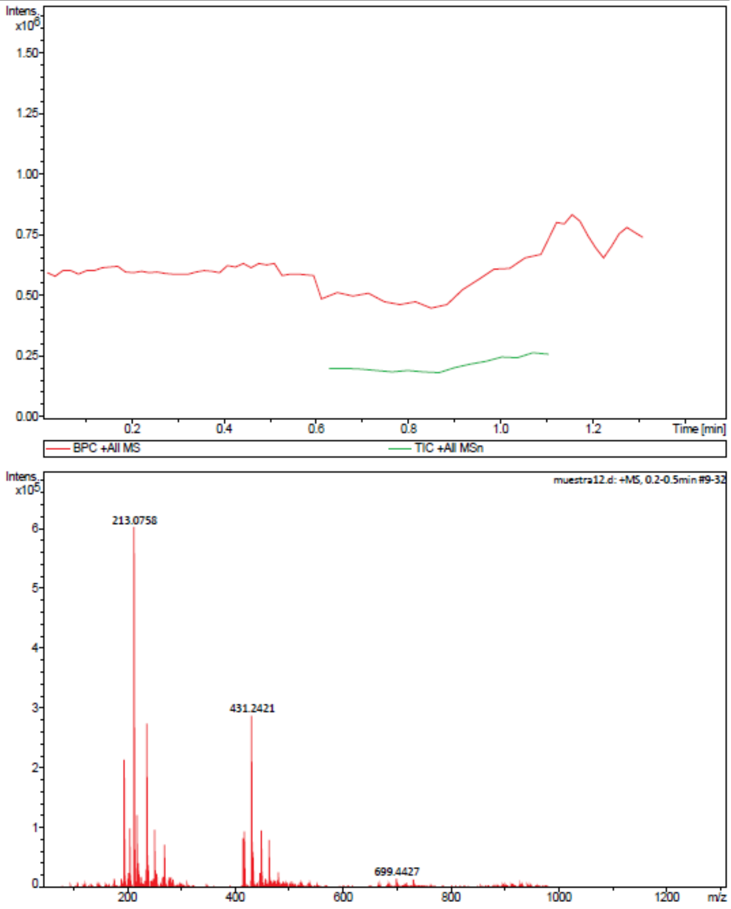

Bruker Compass DataAnalysis 4.4 printed: 20-03-2023 13:06:35 by: Cristian Page 1 of 1

HRMS for **8g**

Generic Display Report

**Analysis Info**		Acquisition Date	15-02-2022 11:04:45
Analysis Name	C:\Users\jgber\Downloads\Datos cuellar\infdir\mc043.d		
Method	DirectInfusion - MS - positive.m	Operator	Demo User
Sample Name	blank	Instrument	compact
Comment			

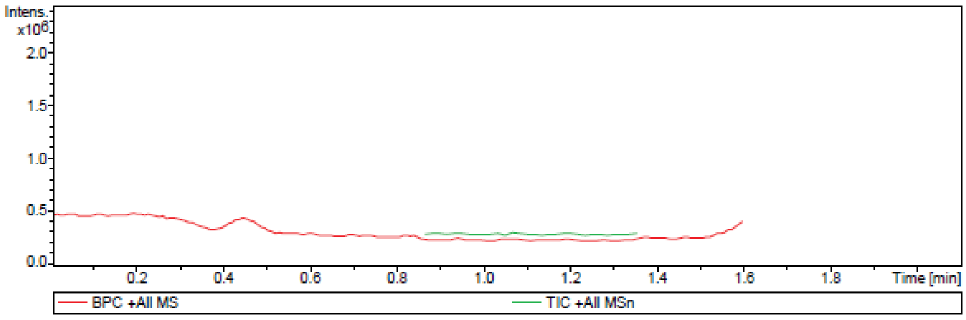

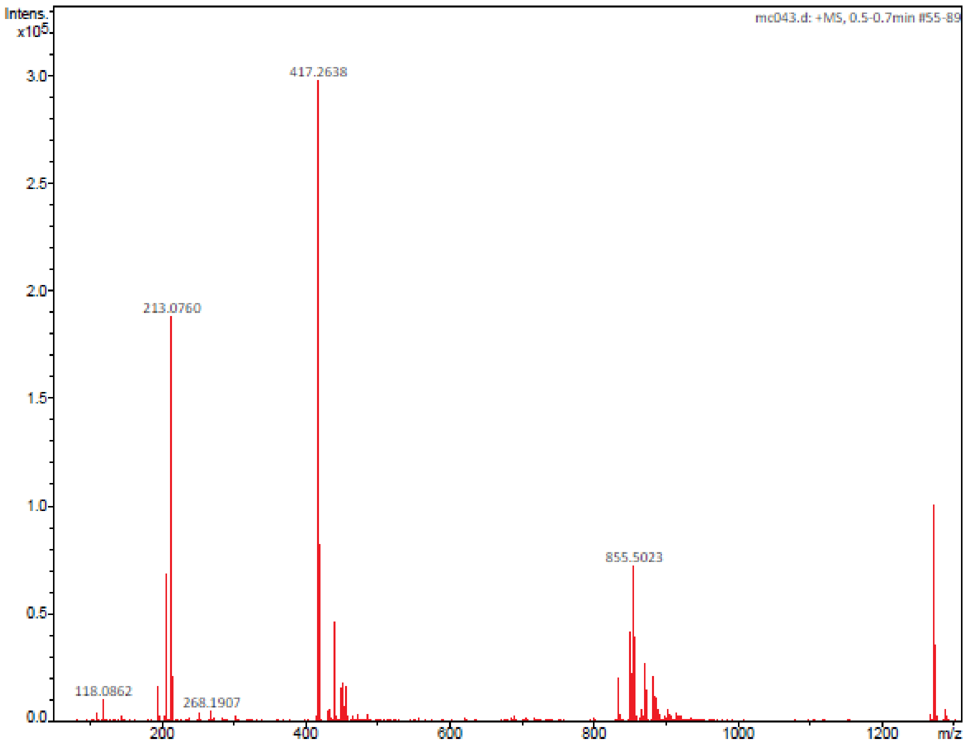

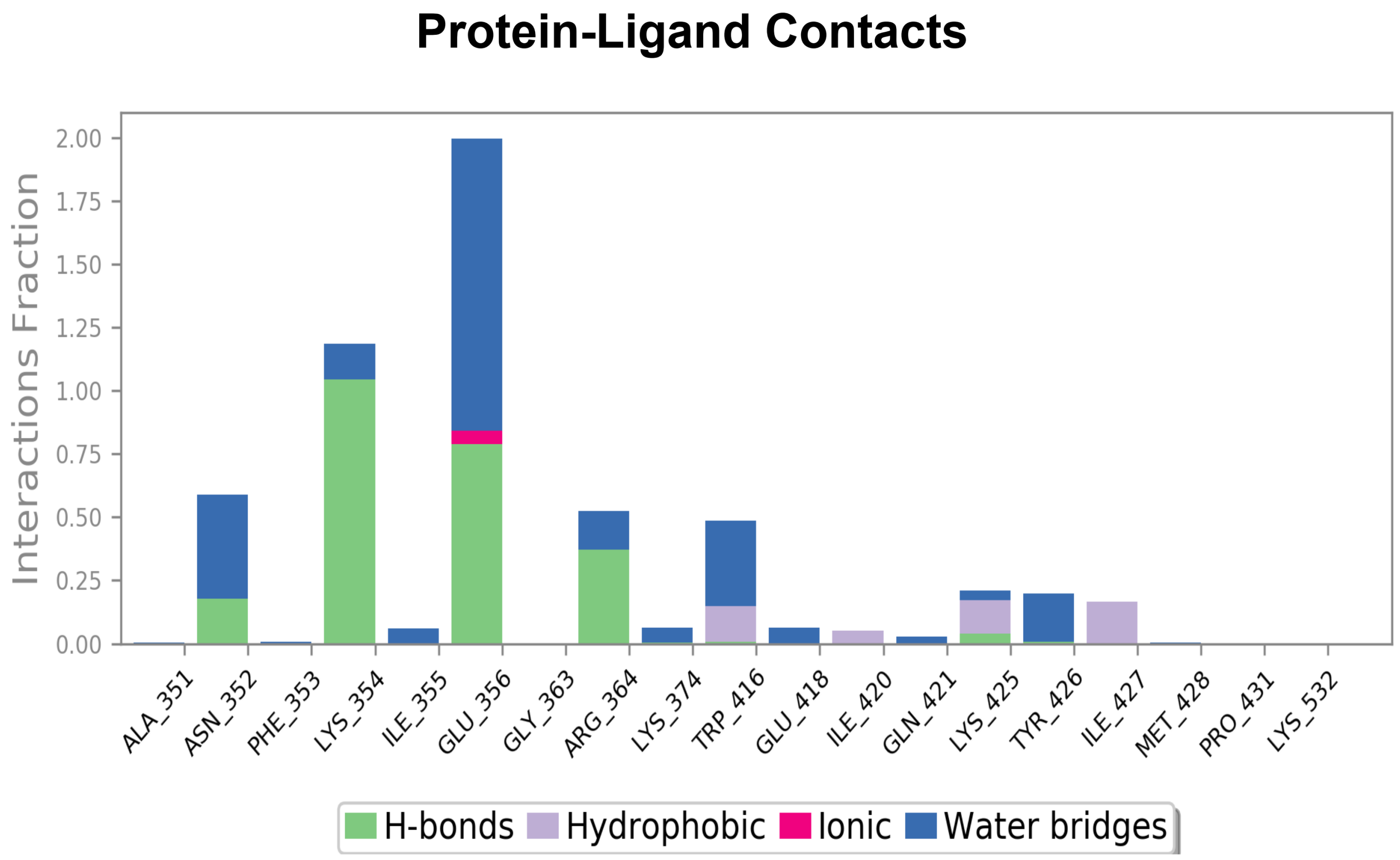

Figure S1. Protein-Daunorubicin contacts over time (100 ns).

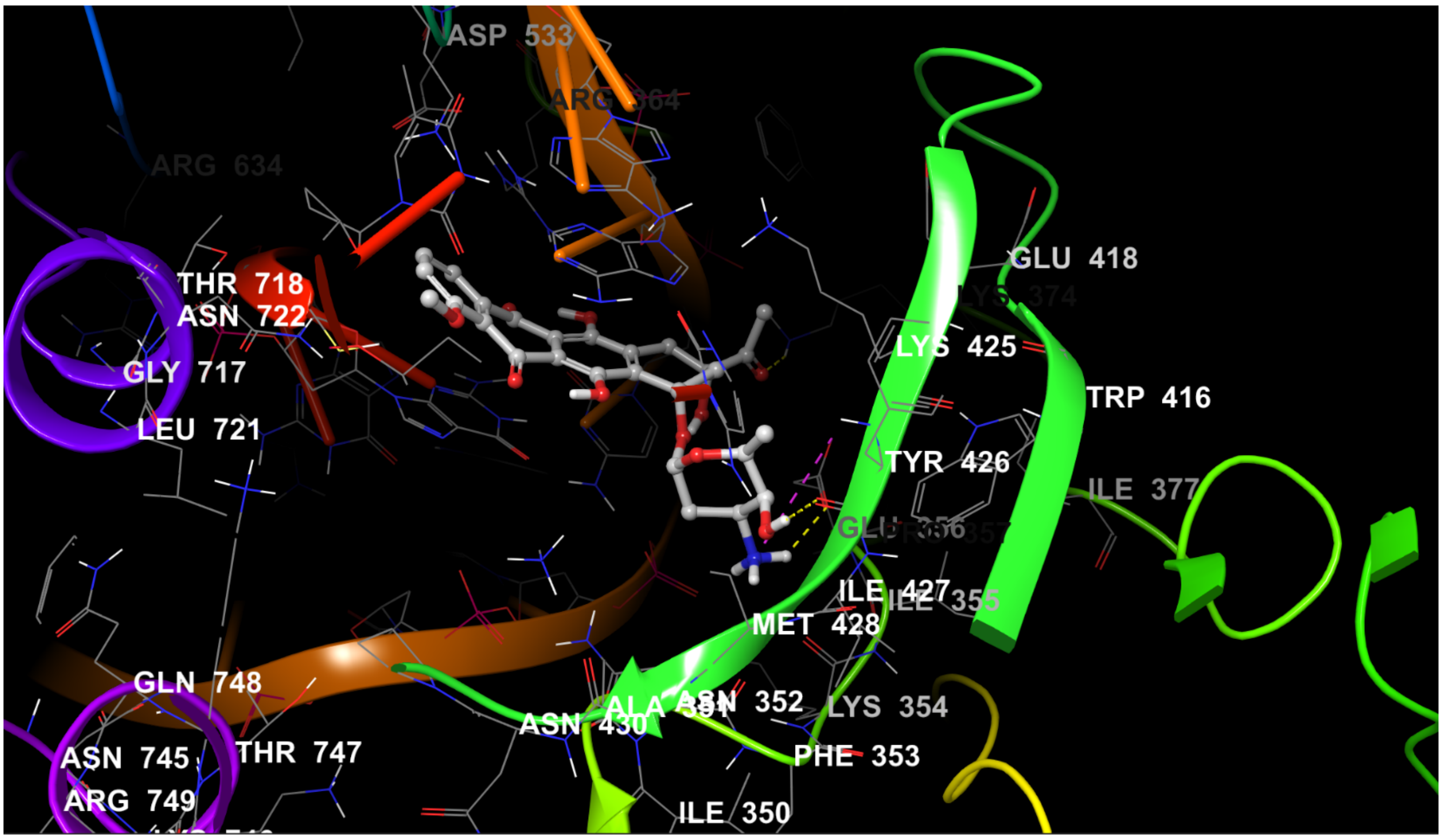

Figure S2. Daunorubicin first frame of molecular dynamics simulation (t =0 ns). Close-view capture. This view is the docking-output pose. The compound accommodates as and intercalating between the nitrogenated bases.

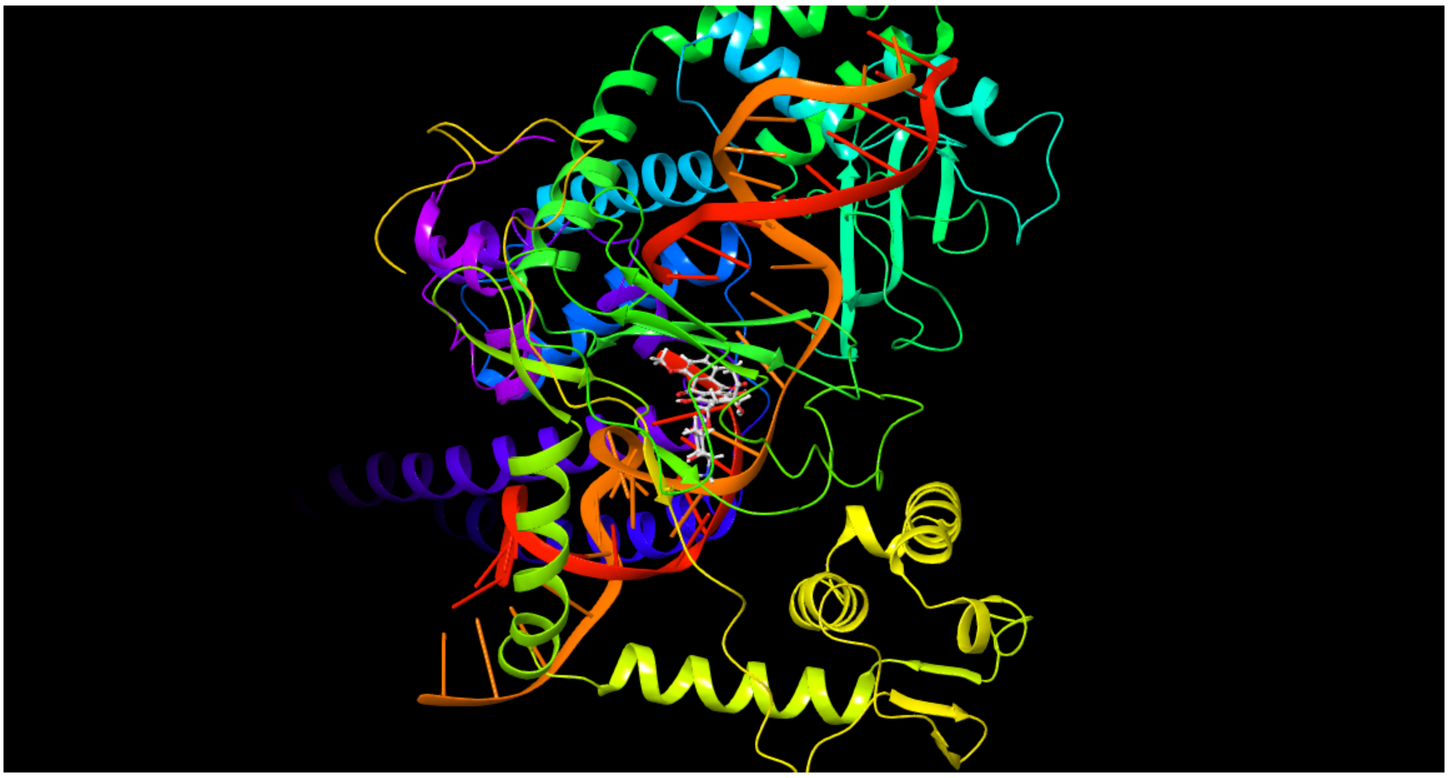

Figure S3. Daunorubicin last frame of molecular dynamics simulation (t = 100 ns). Far-view capture.

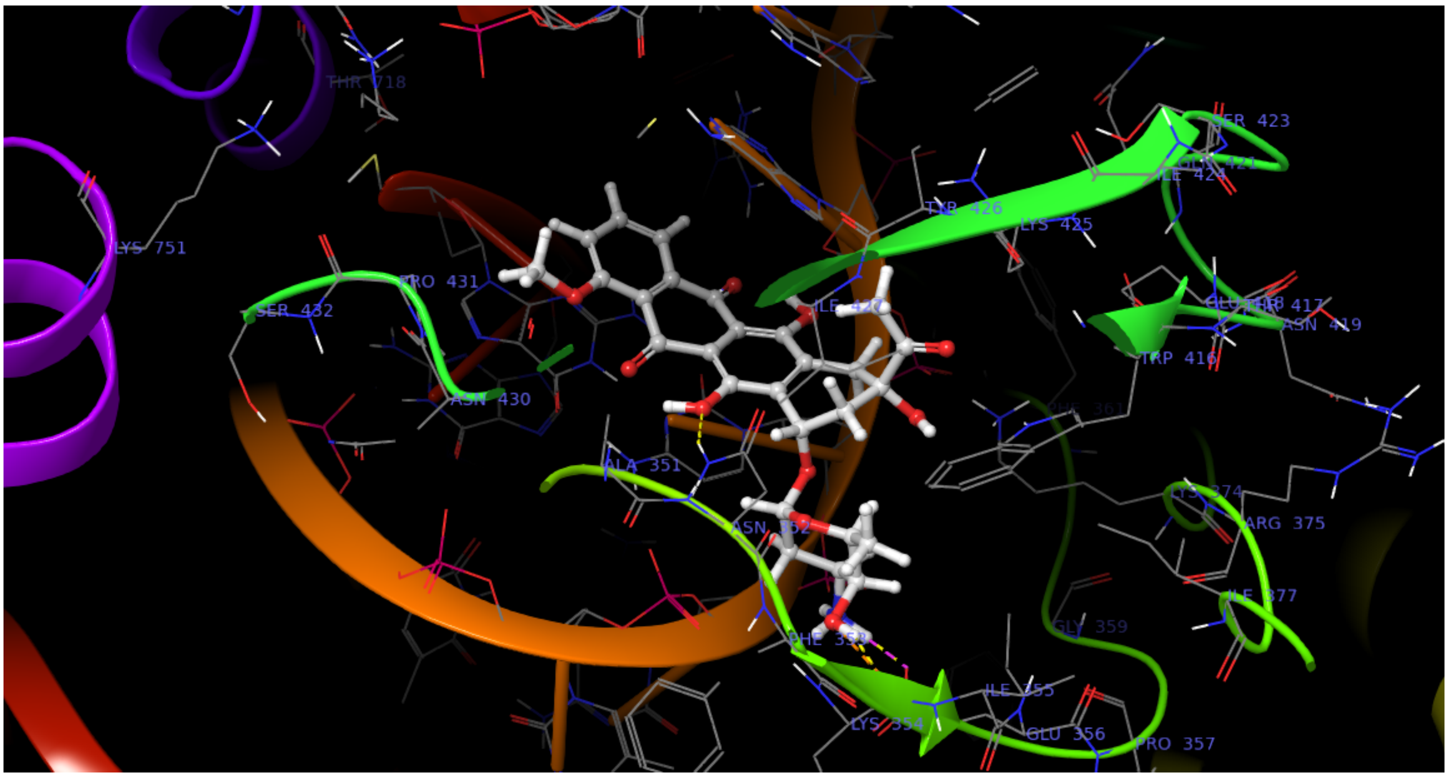

Figure S4. Daunorubicin last frame of molecular dynamics simulation (t= 100 ns). Close-view capture. The compound remains between the nitrogenated bases over time acting as an intercalating-drug.

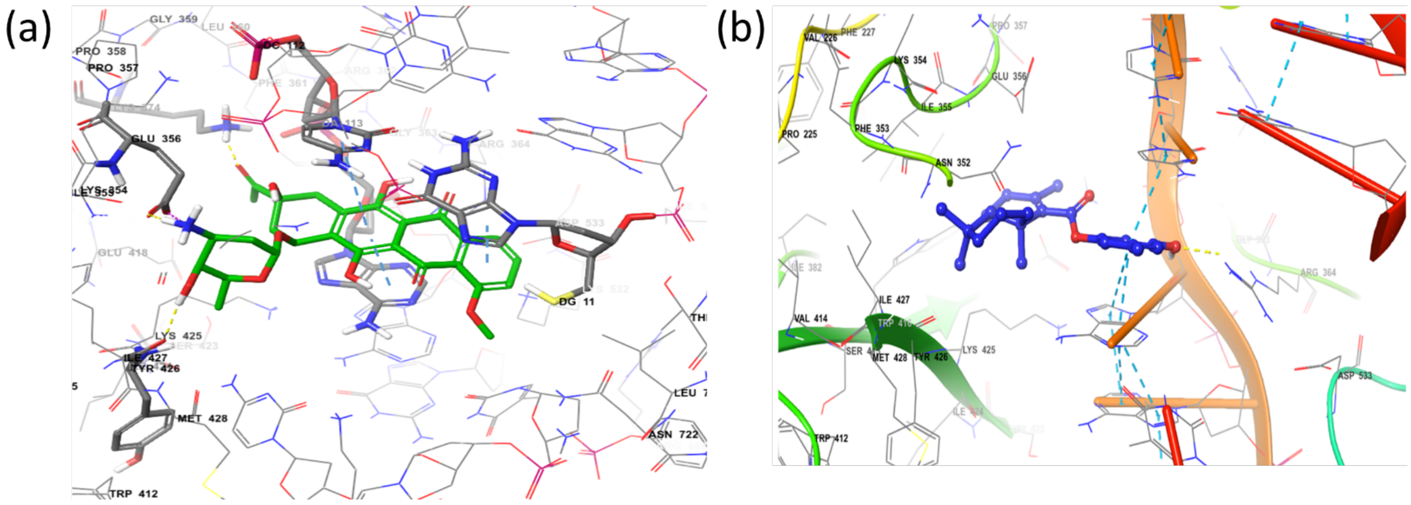

Figure S5. a) 3D pose obtained by docking for daunorubicin at the topotecan reference ligand site. Daunorubicin inserts as an intercalator between the DG11, DC112 and DA113 bases, establishing π-stacking interactions. **b)** 3D pose obtained by docking for compound **6a** at the topotecan reference ligand site. The benzene ring is oriented toward the DNA bases, while the drimanic fragment is oriented towards the enzyme.

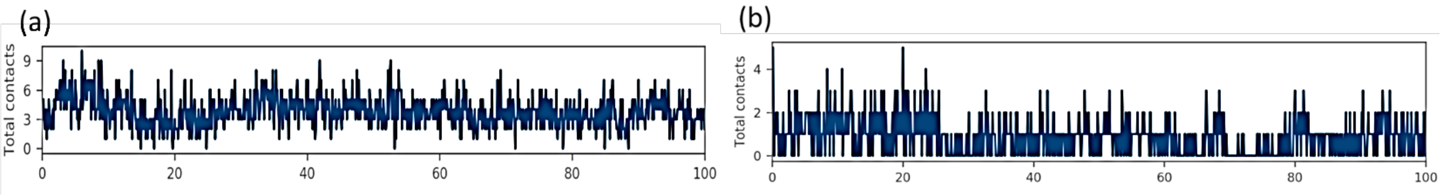

Figure S6. (a, b) Protein-ligand total contacts for daunorubicin, and compound **6a** respectively.
